# Investigations of the CLOCK and BMAL1 Proteins Binding to DNA: A Molecular Dynamics Simulation Study

**DOI:** 10.1371/journal.pone.0155105

**Published:** 2016-05-06

**Authors:** Tuo Xue, Chunnian Song, Qing Wang, Yan Wang, Guangju Chen

**Affiliations:** Key Laboratory of Theoretical and Computational Photochemistry, Ministry of Education, College of Chemistry, Beijing Normal University, Beijing, China; Consejo Superior de Investigaciones Cientificas, SPAIN

## Abstract

The circadian locomotor output cycles kaput (CLOCK), and brain and muscle ARNT-like 1 (BMAL1) proteins are important transcriptional factors of the endogenous circadian clock. The CLOCK and BMAL1 proteins can regulate the transcription-translation activities of the clock-related genes through the DNA binding. The hetero-/homo-dimerization and DNA combination of the CLOCK and BMAL1 proteins play a key role in the positive and negative transcriptional feedback processes. In the present work, we constructed a series of binary and ternary models for the bHLH/bHLH-PAS domains of the CLOCK and BMAL1 proteins, and the DNA molecule, and carried out molecular dynamics simulations, free energy calculations and conformational analysis to explore the interaction properties of the CLOCK and BMAL1 proteins with DNA. The results show that the bHLH domains of CLOCK and BMAL1 can favorably form the heterodimer of the bHLH domains of CLOCK and BMAL1 and the homodimer of the bHLH domains of BMAL1. And both dimers could respectively bind to DNA at its H1-H1 interface. The DNA bindings of the H1 helices in the hetero- and homo-bHLH dimers present the rectangular and diagonal binding modes, respectively. Due to the function of the α-helical forceps in these dimers, the tight gripping of the H1 helices to the major groove of DNA would cause the decrease of interactions at the H1-H2 interfaces in the CLOCK and BMAL1 proteins. The additional PAS domains in the CLOCK and BMAL1 proteins affect insignificantly the interactions of the CLOCK and BMAL1 proteins with the DNA molecule due to the flexible and long loop linkers located at the middle of the PAS and bHLH domains. The present work theoretically explains the interaction mechanisms of the bHLH domains of the CLOCK and BMAL1 proteins with DNA.

## 1. Introduction

The endogenous circadian rhythms in biology as an adaptation to the natural environment allow organisms themselves adapting to the environmental changes, such as temperature and light, in various physiological statuses. The daily sleep-wake rhythm is a well-known circadian rhythm. The metabolic homeostasis is also linked to the circadian rhythms suggested by emerging experiments. Consequently, the disruption of circadian rhythms can lead to body function disorder and diseases, such as sleep disorder, cardiovascular disease, obesity, diabetes and so on [1–6]. The endogenous circadian rhythms driven by the circadian clock in mammals involve negative and positive transcriptional feedback processes regulated by the circadian locomotor output cycles kaput (CLOCK), and brain and muscle ARNT-like 1 (BMAL1) proteins. The CLOCK and BMAL1 proteins can form into a heterodimer, then bind to the specific E-box DNA to activate the transcriptions of the clock-related genes of period (Per), cryptochrome (Cry) and orphan nuclear receptor Rev-Erbα. The translated Per and Cry proteins can reversely act as negative regulators by interacting with the CLOCK and BMAL1 proteins to inhibit the transcriptions of the Per and Cry genes, ending the negative transcriptional feedback process. On the other hand, the inhibition of the translated Rev-Erbα protein for the BMAL1 gene transcription with the auxiliary feedback process could again activate the transcription-translation cycle of the heterodimeric CLOCK and BMAL1 complex binding to E-box DNA, inducing a positive transcriptional feedback process [7–26]. As expected, the transcription-translation activities of these clock-related genes have successfully constructed the molecular basis of circadian clock in mammals. Especially, the hetero-dimerization of the CLOCK and BMAL1 proteins and the combination of heterodimer-DNA play a key role in the positive and negative transcriptional feedback processes. The study on the mechanisms for the dimerization and E-box combination of the CLOCK and BMAL1 proteins will be helpful in better understanding the mechanisms of endogenous circadian clock.

The CLOCK and BMAL1 proteins belonged to the basic helix loop helix—Per Arnt Sim (bHLH-PAS) family of transcriptional regulatory proteins could facilitate the transcriptions of various genes through their bindings to E-box sites [27, 28]. E-box elements can regulate specific gene expression with the specific DNA sequence of CANNTG (where N can be any nucleotides) [29–37]. The palindromic canonical E-box sequence of CACGTG bound by the CLOCK and BMAL1 proteins has been investigated by Charles J. Weitz and co-workers in 1998 [9]. Each of the CLOCK and BMAL1 proteins consists of one bHLH domain, one PAS domain, one C-terminal region and some loop linkers [15, 21]. Namely, a bHLH domain with the ~50 amino acids is constructed by two α-helices (as H1 and H2) and one loop linker [38]. A PAS domain with the 260~310 amino acids is subdivided into two well-conserved PAS-A and PAS-B domains, and a loop linker [27]. The structures of the bHLH and PAS domains in CLOCK and BMAL1 are similar to those in the aryl hydrocarbon receptor nuclear transporter (ARNT), dioxin receptor (DR) and hypoxia inducible factor 1 (HIF-1) proteins that have been extensively investigated by a lot of experiments. For example, it has been reported by Richard G. Brennan and co-workers in 2001 that the ARNT-bHLH peptides can form homodimers that can bind E-box DNA with high affinity under the low concentration *in vitro* [39]. Kevin H. Gardner and co-workers revealed that the PAS domains of ARNT can form homo- and hetero-dimers by using of a common beta-sheet interface in 2005 [40]. In 2004, Anne Chapman-Smith and co-workers studied the formation of stable protein-DNA complexes by DR/ARNT and HIF-1/ARNT heterodimers with their cognate DNA sequences [41]. Moreover, it has been found that the mouse Per, CLOCK, and BMAL1 proteins undergo robust circadian changes in phosphorylation [42]. Joseph S. Takahashi and co-workers first reported the X-ray crystal structure of *Mus musculus* bHLH-PAS domains of CLOCK:BMAL1 heterodimer in 2012 [43]. They also investigated the effect of some mutations at the CLOCK and BMAL1 heterodimer interfaces on the periodicity of the circadian oscillator, and on the stability and activity of the CLOCK:BMAL1 complex. Later on, Xiao-Dong Su and co-workers reported the X-ray crystal structure of the hetero-complex: *Homo sapiens* bHLH domains of CLOCK:BMAL1 heterodimer binding to E-box DNA [44]. It was predicted that the individual CLOCK or BMAL1 bHLH domain can also form a homodimer structure in solution. And the mutual recognition mechanism of the bHLH domains of the CLOCK and BMAL1 heterodimer has been concluded through the homodimer-mimicking experiment [44]. It has been found that the computational and mathematical modeling was used to investigate cellular rhythms that involve the CLOCK and BMAL1 proteins [45–47]. For example, it has been suggested based on the experimental data that the increase of the amplitude of circadian oscillations can enhance immunity to molecular noise using the stochastic mathematical model of the mammalian circadian clock [45]. Some similar structural proteins, such as the photoactive yellow protein (PYP), inhibitor of differentiation 3 (ID-3) and twist-related protein 1 (TWIST1) were theoretically investigated [48–58]. Homology modeling and molecular dynamics simulations were employed to study the folding and unfolding characteristics of the PYP protein [49–52, 54]. Monte Carlo simulation was carried out to study the unfolding pathways of the PAS-B domain of ARNT protein [48]. The dimerization and DNA-recognition properties for the HIF-1 and TWIST1 proteins were revealed by modeling and molecular simulations [55–58]. However, theoretical studies on the structural characters for the CLOCK and BMAL1 proteins at the atomic level are scarce so far. Especially, detail informations about hetero-/homo-dimerization, protein-DNA affinities, and DNA-binding mechanisms for the CLOCK and BMAL1 proteins are necessary to better understand their regulation mechanisms for the transcription-translation activities of the clock-related genes through DNA binding.

To elucidate the dimerization of the bHLH-PAS domains of CLOCK and BMAL1, and DNA combination, we carried out molecular dynamics (MD) simulations and free energy calculations for some binary and ternary models of the bHLH/bHLH-PAS domains of the CLOCK and BMAL1 proteins, and the DNA molecule. Three MD simulations were performed on the hetero- and homo-dimers of the bHLH domains of CLOCK and BMAL1 to investigate the dimerization characteristics of the bHLH domains. Two simulations were performed on the ternary complexes of the bHLH domains of CLOCK and BMAL1 binding to the DNA molecule to address the bHLH-DNA binding mechanisms. More simulations were performed on the corresponding phosphorylated proteins and the PAS domains of the proteins to study the influences of phosphorylation and PAS domains on DNA binding. This study would help people to understand the regulation mechanism of endogenous circadian clock affected by the heterodimeric CLOCK and BMAL1 proteins.

## 2. Materials and Methods

### 2.1. Initial structures

**Fig 1:**
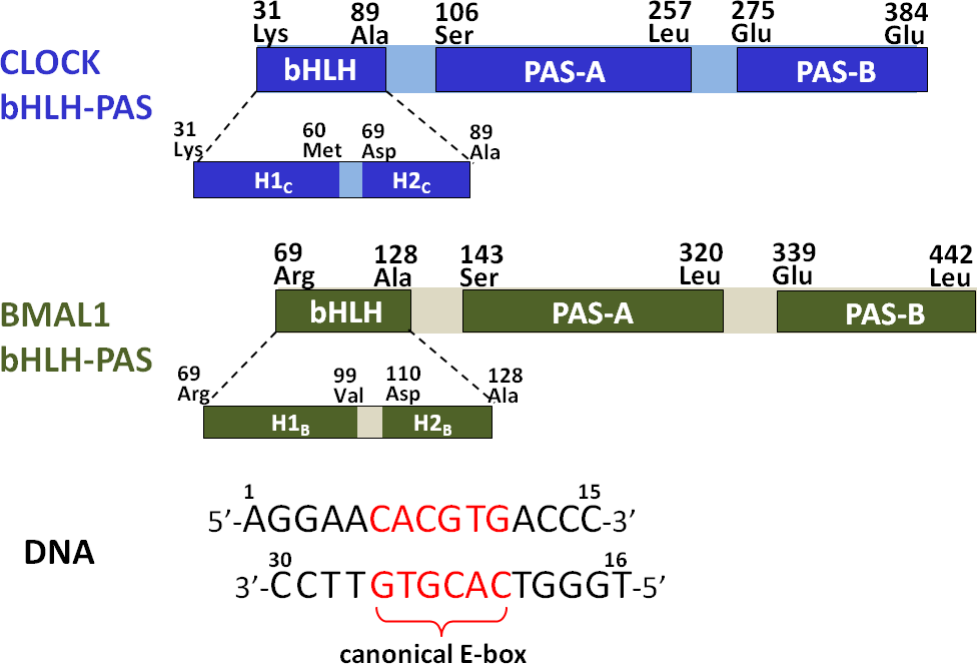
Compositions of the bHLH-PAS domain of CLOCK/BMAL1 protein, and DNA. The amino acid extremities and the domain organization of the bHLH-PAS domains of the CLOCK and BMAL1 proteins, and the base sequence of DNA.

Based on the previous experimental studies [15, 21], the bHLH-PAS domain in the CLOCK or BMAL1 protein consists of the bHLH, PAS-A and PAS-B domains, and two loop linkers. A bHLH domain is further subdivided into two helical regions (assigned as H1_C_ and H2_C_ for the CLOCK protein, and H1_B_ and H2_B_ for the BMAL1 protein). The amino acid extremities and the domain organization of the bHLH-PAS domains in the CLOCK (UniProtKB/Swiss-Prot: O15516.1) and BMAL1 (UniProtKB/Swiss-Prot: O00327.2) proteins of *Homo sapiens*, and the base sequence of E-box DNA used in this work are shown in Fig 1. The structure of the heteromeric CLOCK bHLH + BMAL1 bHLH + DNA complex of *Homo sapiens* as the starting structure for the MD simulation was taken from its X-ray structure (PDB entry 4H10) (assigned as C_bHLH_+B_bHLH_+DNA model) [44]. Based on the structure of the C_bHLH_+B_bHLH_+DNA model, the structure of the homomeric BMAL1 bHLH + BMAL1 bHLH + DNA complex was constructed by substituting the bHLH domain of the BMAL1 protein for one of the CLOCK protein (assigned as B_bHLH_+B_bHLH_+DNA model). Namely, the structure of the bHLH domain of the BMAL1 protein was taken from that in the C_bHLH_+B_bHLH_+DNA model, followed by directly superposing the obtained structure of the bHLH domain of BMAL1 onto that of CLOCK in the C_bHLH_+B_bHLH_+DNA model, then deleting the extra coordinates of the bHLH domain of CLOCK, and importing all the coordinates of the B_bHLH_+B_bHLH_+DNA model using the PyMol program (http://www.pymol.org). The structures of the heterodimeric CLOCK bHLH + BMAL1 bHLH complex, and the homodimeric BMAL1 bHLH + BMAL1 bHLH complex were constructed by deleting the DNA coordinates in the C_bHLH_+B_bHLH_+DNA and B_bHLH_+B_bHLH_+DNA models, respectively (assigned as C_bHLH_+B_bHLH_ and B_bHLH_+B_bHLH_ models). The structure of the homodimeric CLOCK bHLH + CLOCK bHLH complex was constructed by using the similar constructing method for the B_bHLH_+B_bHLH_+DNA model with the superposing of the structure of the bHLH domain of CLOCK onto that of the BMAL1 protein in the C_bHLH_+B_bHLH_+DNA model, followed by deleting the DNA coordinates (assigned as C_bHLH_+C_bHLH_ model). In order to investigate the binding property of protein-DNA influenced by phosphorylation, the phosphorylated C_bHLH_+B_Phos_+DNA and B_Phos_+B_Phos_+DNA models with the phosphorylation of Ser78 (Ser(PO3)78) at the H1_B_ helix were constructed through modifying the C_bHLH_+B_bHLH_+DNA and B_bHLH_+B_bHLH_+DNA models. That is, the residue Ser(PO3)78 was built using the TLEAP module in AMBER9 program [59]. The parameters of Ser(PO3)78 were referenced from the previous works [60, 61]. Details of the construction procedures of the phosphorylated models can be found in S1 Text of the Supporting Information. Moreover, the heterodimeric model with the PAS and bHLH domains of the CLOCK and BMAL1 proteins, assigned as C_bHLH_+B_bHLH_+PAS model, was constructed by modifying the X-ray crystal structure of CLOCK bHLH-PAS + BMAL1 bHLH-PAS complex (PDB entry 4F3L) from *Mus musculus* to *Homo sapiens* with 99% sequence identity by using the homology modeling technologies in the “Build Mutants protocol” of the Discovery Studio visualizer (http://accelrys.com/). And the missing residues in this model were repaired using the loop search method in the Swiss-Pdb Viewer (http://spdbv.vital-it.ch/) [43, 62]. Similarly, the corresponding heterotrimeric C_bHLH_+B_bHLH_+PAS+DNA model was constructed by directly superposing the C_bHLH_+B_bHLH_+DNA model onto the C_bHLH_+B_bHLH_+PAS model, then deleting the extra coordinates of the bHLH domains of CLOCK and BMAL1 in the C_bHLH_+B_bHLH_+DNA model, and importing all the coordinates of the C_bHLH_+B_bHLH_+PAS+DNA model. To compare the differences of conformations between the disturbed DNA molecule and a canonical DNA molecule, a canonical B-DNA molecule simulation was also performed. 60 Na^+^, 48 Cl^-^ and 48 Na^+^, 67 Cl^-^ counterions for the ternary C_bHLH_+B_bHLH_+DNA model and the binary C_bHLH_+B_bHLH_ model, respectively, were added to achieve electroneutrality and to satisfy the experimental ionic strength of 200mM [44]. Similar counterion processes were applied to other models. The systems were explicitly solvated by using the transferable intermolecular potential 3P water inside a rectangular box large enough to ensure the solvent shell extended to 8 Å in all directions of each system studied.

### 2.2. Molecular dynamics simulation protocols

All MD simulations for the nine models were carried out using the AMBER9 package [59] with a classical AMBER parm99 force field [63, 64], and the parmbsc0 refinement [65] and gaff force field parameters [66]. To test the structural convergence, three independent MD simulations for each of the three binary C_bHLH_+B_bHLH_, B_bHLH_+B_bHLH_ and C_bHLH_+C_bHLH_ models were performed. Based on the starting structure referenced from the X-ray structure and the same counterion strength, the only difference in the three independent MD simulations for each model was the different starting velocities usually randomly assigned in the simulation running. The computational details and the corresponding results can be found in S1 Text, and S1A and S1B Fig of the Supporting Information.

### 2.3. Binding free energy analyses

The molecular mechanics Poisson-Boltzmann surface area (MM-PBSA) method in AMBER9 package was employed to perform the binding free energy analyses [67–70]. The binding free energy (Δ*G*_binding_) was computed through calculating the free energy differences of ligand, receptor and their complex as follows:

$$\Delta G_{binding}=G_{complex}-G_{ligand}-G_{receptors}$$

where *G*_complex_, *G*_ligand_, and *G*_receptors_ are the free energies of complex, ligand and receptor, respectively. To verify the accuracy of the calculated energies, the protein-protein MM-PBSA binding free energies for the C_bHLH_+B_bHLH_ model were calculated from three independent MD simulations by extracting the last 10ns trajectories. The computational details and the corresponding results are available in S2 Text and S1 Table of the Supporting Information, and our previous studies [71, 72].

### 2.4. Analyses of fluctuation, correlation, interaction, interhelical angle/distance, and DNA groove parameter

The root-mean-square fluctuations (RMSF) values, correlation of motions, hydrogen bond/hydrophobic interaction, interhelical angle/distance, and DNA groove parameter were calculated by using PTRAJ module in AMBER9 program [59], INTERHLX [73, 74] and CURVES programs [75]. Computational details are available in S3 Text of the Supporting Information.

## 3. Results

**Fig 2:**
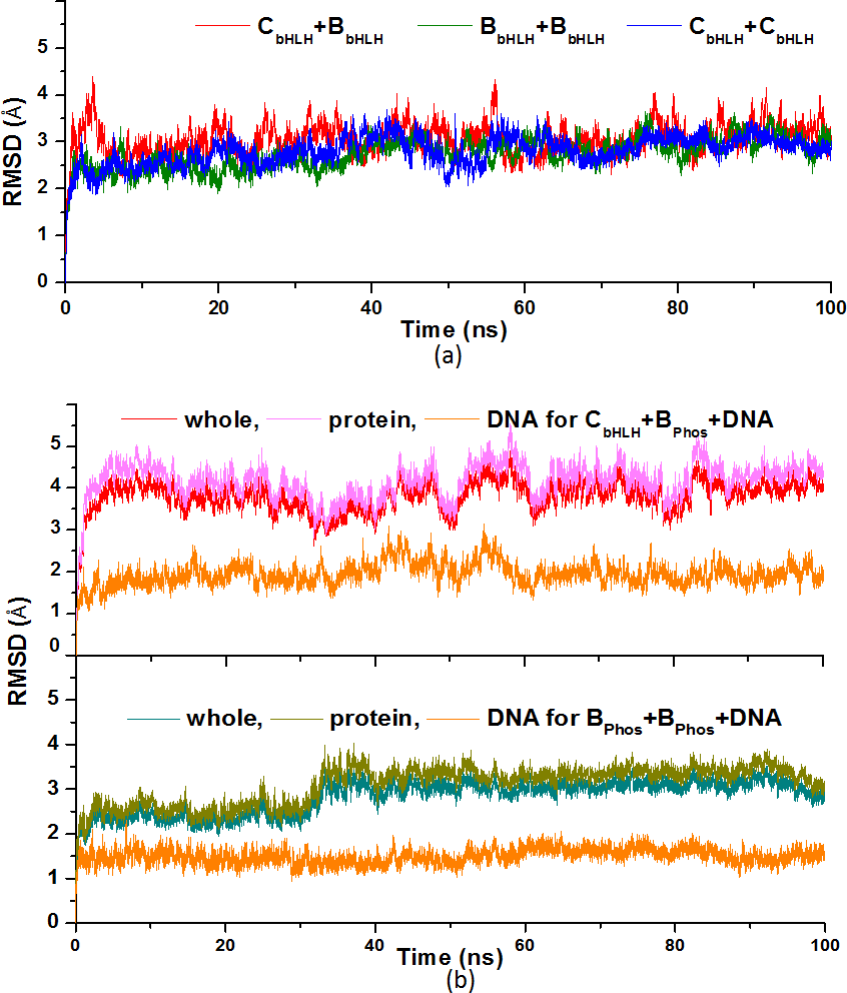
RMSD values of the binary and phosphorylated models. RMSD values of all heavy atoms with respect to the corresponding starting structures for MD simulations of (a) the C_bHLH_+B_bHLH_, B_bHLH_+B_bHLH_ and C_bHLH_+C_bHLH_ models, and (b) the C_bHLH_+B_Phos_+DNA and B_Phos_+B_Phos_+DNA models.

**Fig 3:**
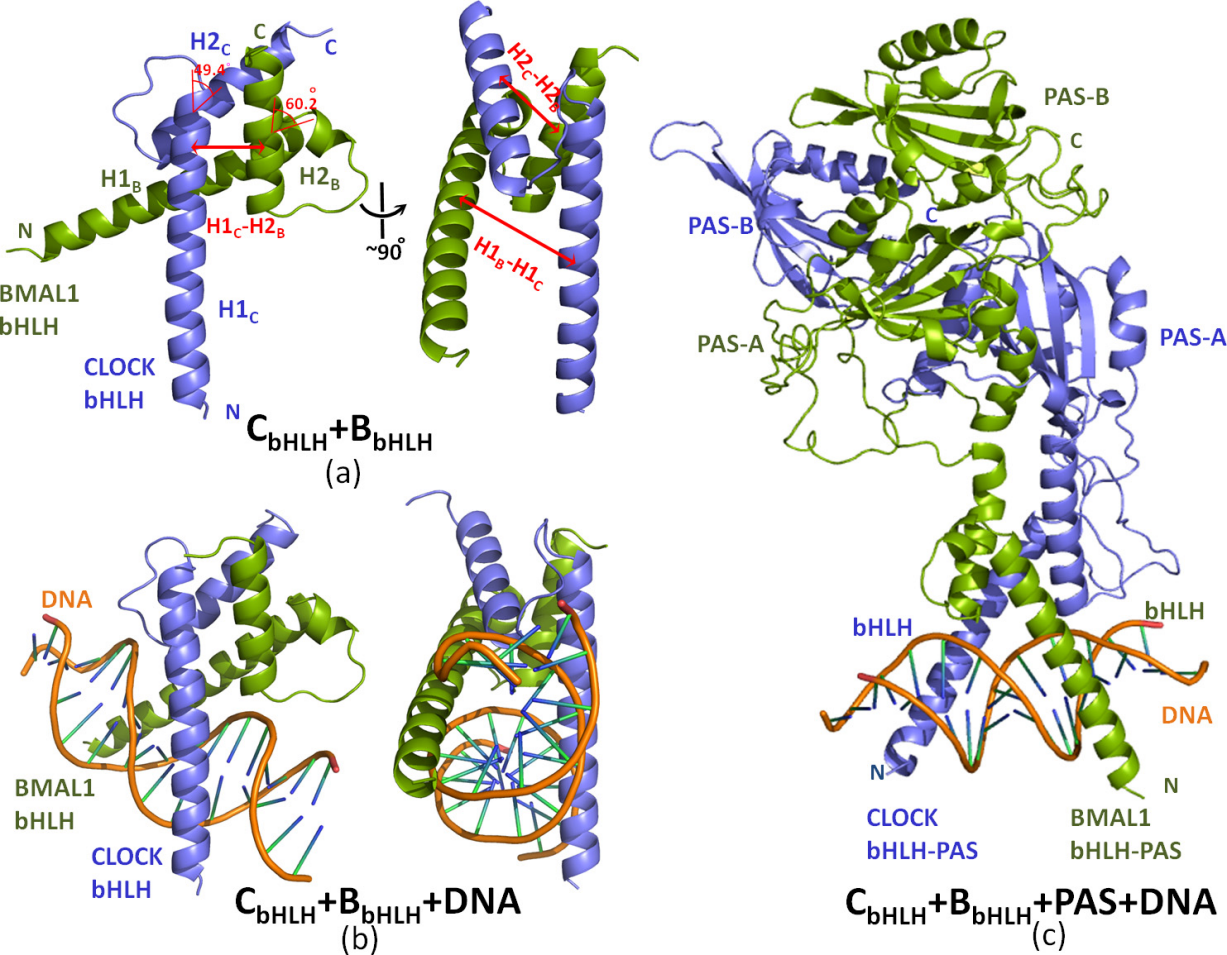
Structures of three models for the CLOCK and BMAL1 proteins, and DNA. Average simulated structures for (a) the C_bHLH_+B_bHLH_ model, (b) the C_bHLH_+B_bHLH_+DNA model, and (c) the C_bHLH_+B_bHLH_+PAS+DNA model.

To explore the combination properties of the CLOCK and BMAL1 proteins with DNA, the MD simulations for four binary C_bHLH_+B_bHLH_, B_bHLH_+B_bHLH_, C_bHLH_+C_bHLH_, C_bHLH_+B_bHLH_+PAS models, and five ternary C_bHLH_+B_bHLH_+DNA, B_bHLH_+B_bHLH_+DNA, C_bHLH_+B_Phos_+DNA, B_Phos_+B_Phos_+DNA, C_bHLH_+B_bHLH_+PAS+DNA models were performed with explicit water and certain counterions. Namely, the 100ns MD simulations for the small binary C_bHLH_+B_bHLH_, B_bHLH_+B_bHLH_, C_bHLH_+C_bHLH_, and the phosphorylated C_bHLH_+B_Phos_+DNA and B_Phos_+B_Phos_+DNA models, and the 50ns simulations for other models were performed due to the computational cost. The root-mean-square deviation (RMSD) values of the heavy atoms were calculated referenced to the experimental crystal structure for the C_bHLH_+B_bHLH_+DNA model, and to the corresponding starting structures for other models over the courses of the trajectories. The very flexible residues Lys31-Asn40 for the CLOCK protein and Arg69-Ser78 for the BMAL1 protein were omitted from the RMSD analysis because they caused high RMSD values that are not indicative of any significant structural changes of interest in the C_bHLH_+B_bHLH_, B_bHLH_+B_bHLH_ and C_bHLH_+C_bHLH_ models. The corresponding results for three binary C_bHLH_+B_bHLH_, B_bHLH_+B_bHLH_ and C_bHLH_+C_bHLH_ models, and the phosphorylated C_bHLH_+B_Phos_+DNA and B_Phos_+B_Phos_+DNA models are plotted in Fig 2A and 2B, respectively. Other results for other models are shown in S2A–S2C Fig of the Supporting Information. It is often considered that small RMSD values of one simulation indicate a stable state of the system, and also suggest that the newly constructed models in this work can satisfactorily reproduce the experimental structures. It can be seen that the C_bHLH_+B_bHLH_, B_bHLH_+B_bHLH_, C_bHLH_+C_bHLH_, C_bHLH_+B_bHLH_+DNA, B_bHLH_+B_bHLH_+DNA, C_bHLH_+B_bHLH_+PAS and C_bHLH_+B_bHLH_+PAS+DNA models reached equilibrium after 30 ns, and their energies were found to be stable during the remainder of each simulation. Therefore, the equilibrated conformation for each system was extracted from the trajectory analysis of the last 10ns equilibrium simulation, recording 5000 snapshots at every 2ps time-interval of each trajectory. However, the equilibrated conformations for the C_bHLH_+B_Phos_+DNA and B_Phos_+B_Phos_+DNA models with the phosphorylated residue Ser78 of the BMAL1 protein were extracted from the last 20 ns of equilibrium simulation time, recording 10000 snapshots at every 2ps time-interval due to the unstable characteristics of the phosphorylated BMAL1 protein binding to DNA. The average structures for only C_bHLH_+B_bHLH_, C_bHLH_+B_bHLH_+DNA and C_bHLH_+B_bHLH_+PAS+DNA models are depicted in Fig 3A–3C, respectively.

### 3.1. Stability of the binary C_bHLH_+B_bHLH_, B_bHLH_+B_bHLH_ and C_bHLH_+C_bHLH_ models

#### 3.1.1. Binding free energy analysis

To address the binding properties between the bHLH domains in the CLOCK and BMAL1 proteins, the binding free energies of the C_bHLH_+B_bHLH_, B_bHLH_+B_bHLH_ and C_bHLH_+C_bHLH_ models were calculated by using the MM-PBSA methodology, and are shown in Table 1 with all energy terms. It can be seen that the binding free energies between the bHLH domains are -12.55, -14.11 and 13.21 kcal·mol^-1^ for the C_bHLH_+B_bHLH_, B_bHLH_+B_bHLH_ and C_bHLH_+C_bHLH_ models, respectively, which indicate that the C_bHLH_+B_bHLH_ and B_bHLH_+B_bHLH_ models are more stable than the C_bHLH_+C_bHLH_ model. Particularly, the binding free energy of -12.55 kcal·mol^-1^ for the C_bHLH_+B_bHLH_ model is comparable with the experimental result of -8.77 kcal·mol^-1^ from the isothermal titration calorimetry measurements [44]. The present results indicate that the dimers of C_bHLH_+B_bHLH_ and B_bHLH_+B_bHLH_ are energetically favorable *in vivo*, and expectedly, also support the experimentally mutual recognition mechanism between two bHLH domains of CLOCK and BMAL1 [44].

**Table 1:** Components of the MM-PBSA free energies (kcal·mol^-1^) for the binary C_bHLH_+B_bHLH_, B_bHLH_+B_bHLH_, and C_bHLH_+C_bHLH_ models, and the ternary C_bHLH_+B_bHLH_+DNA and B_bHLH_+B_bHLH_+DNA models.

	C_bHLH_+B_bHLH_	B_bHLH_+B_bHLH_	C_bHLH_+C_bHLH_	C_bHLH_+B_bHLH_+DNA		B_bHLH_+B_bHLH_+DNA	
Receptor	C_bHLH_	B_bHLH_	C_bHLH_	C_bHLH_	C_bHLH_+B_bHLH_	B_bHLH_	B_bHLH_+B_bHLH_
Ligand	B_bHLH_	B_bHLH_	C_bHLH_	B_bHLH_	DNA	B_bHLH_	DNA
ΔE_ele_	929.54	637.15	1484.86	1115.98	-10442.97	811.08	-8618.15
ΔE_vdw_	-106.90	-107.50	-101.85	-105.21	-143.64	-103.73	-124.64
Δ*E*_int_	0.00	0.00	0.00	0.00	0.00	0.00	0.00
ΔG_np/solv_	-13.25	-14.03	-13.06	-12.88	-18.39	-12.93	-16.61
ΔG_pb/solv_	-899.63	-630.46	-1435.39	-1071.68	10398.74	-761.88	8568.71
ΔG_np_	-120.21	-121.53	-114.91	-118.09	-162.03	-116.56	-141.25
ΔG_pb_	43.16	36.72	62.52	57.17	-44.23	49.18	-32.83
ΔTS	-64.50	-70.70	-65.59	-60.79	-107.62	-67.49	-95.32
ΔH_binding_	-77.05	-84.81	-52.38	-60.92	-206.26	-67.38	-174.08
ΔG_binding_	-12.55	-14.11	13.21	-0.13	-98.64	0.11	-78.76

Δ*G*_np_ = Δ*E*_vdw_ + Δ*G*_np/solv_, Δ*G*_pb_ = Δ*E*_ele_ + Δ*G*_pb/solv_

ΔG_binding_ = ΔG_np_ + ΔG_pb_− ΔTS = ΔH_binding_− ΔTS.

#### 3.1.2. Conformation and interaction analyses

**Fig 4:**
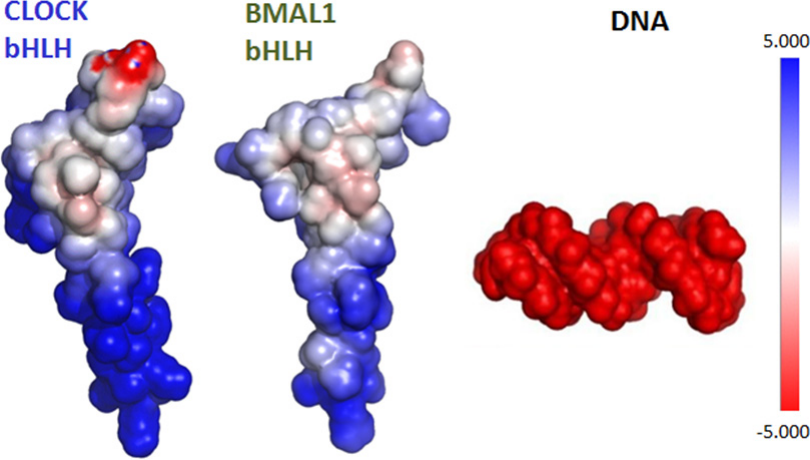
The electrostatic surface potentials of the C_bHLH_+B_bHLH_+DNA model. The electrostatic surface potentials for the bHLH domains of the CLOCK and BMAL1 proteins, and for the DNA molecule in the C_bHLH_+B_bHLH_+DNA model.

It can be seen from Fig 3A that the bHLH domain of CLOCK (slate blue)/BMAL1 (olive green) in the C_bHLH_+B_bHLH_ model consists of a lengthy N-terminal α-helix H1_C/B_ and a short α-helix H2_C/B_ separated by a linker into two layers with their interhelical angle of 49.4/60.2°. The two monomers of the bHLH domains intertwine together, and form into an asymmetry structure of four-helical cross-bundle with the two face-to-face frames of the H1/2_C_ helix of CLOCK (slate blue) and the H1/2_B_ helix of BMAL1 (olive green), and two parallel-side frames of the H1_C/B_ and H2_B/C_ helices by each other. The helical distances between the H1_C_ and H1_B_ helices, and between the H2_C_ and H2_B_ helices, and the average helical distance between the H1_C/B_ and H2_B/C_ helices are respectively 18.5 Å, 12.5 Å and 10.5 Å. Therefore, the contribution of the protein-protein interaction mainly comes from the parallel-side H1_C/B_-H2_B/C_ interfaces of the hetero-bHLH domains in the C_bHLH_+B_bHLH_ model. The forceps structure of the H1_C_ and H1_B_ helices from the half of four-helical cross-bundle of the hetero-bHLH domains contributes to bind towards the major groove of DNA. Similar structure characters were also found in the B_bHLH_+B_bHLH_ and C_bHLH_+C_bHLH_ models. Note that the symmetry of the four-helical cross-bundle structure was not found in the homodimeric B_bHLH_+B_bHLH_ and C_bHLH_+C_bHLH_ models due to the flexibility of the H1_B/C_ helix to bind suitably to DNA [56]. To explore the interactions between the bHLH domains of CLOCK and BMAL1, the percentages of occurrences of all possible hydrogen bonds and hydrophobic interactions located at the H1_C/B_-H2_B/C_ and H1_B/C_-H2_B/C_ interfaces for the C_bHLH_+B_bHLH_, B_bHLH_+B_bHLH_ and C_bHLH_+C_bHLH_ models extracted from the MD trajectories were analyzed, and are listed in Table 2 (The corresponding calculation details are given in S3 Text of the Supporting Information). There are lots of the hydrogen bonds and the hydrophobic interactions at the H1_C/B_-H2_B/C_ and H1_B_-H2_B_ interfaces for the C_bHLH_+B_bHLH_ and B_bHLH_+B_bHLH_ models. However, only hydrophobic interactions at the H1_C_-H2_C_ interfaces were found for the C_bHLH_+C_bHLH_ model. For example, the hydrophobic analysis predicts that two hydrophobic interactions between the two C atoms of Met60 in CLOCK and Met122 in BMAL1 at the H1_C_-H2_B_ interface, and between that of Ile78 in CLOCK and Leu98 in BMAL1 at the H1_B_-H2_C_ interface in the C_bHLH_+B_bHLH_ model spend 84% and 90% of their simulation times, respectively (see Table 2). It was noted that the stable dimers of the bHLH domains of the CLOCK and BMAL1 proteins are mainly driven by more hydrophobic contacts over less hydrogen bonds at the H1_C/B_-H2_B/C_ interfaces, which has been experimentally discussed for the bHLH domain family proteins [76]. However, only one strong hydrophobic interaction between Ser77 in CLOCK and Met122 in BMAL1 located at the face-to-face H2_C_-H2_B_ interface with the occupation of 79% during the simulation time was found in the C_bHLH_+B_bHLH_ model, which is consistent with the experimental prediction from the mutually recognition mechanism [44]. These results support the main contribution of the interactions at the parallel-side H1_C/B_-H2_B/C_ interfaces. The considerable quantities of hydrogen bonds and hydrophobic interactions in the C_bHLH_+B_bHLH_ and B_bHLH_+B_bHLH_ models over that in the C_bHLH_+C_bHLH_ model support the stability of the C_bHLH_+B_bHLH_ and B_bHLH_+B_bHLH_ models. Additionally, the electrostatic surface potentials of the bHLH domains in CLOCK and BMAL1, and the DNA molecule were calculated using the adaptive Poisson-Bolzmann solver (APBS) PyMol plug-in, and are shown in Fig 4 [77]. It can be revealed that the electrostatic repulsion at the H1_C_-H2_C_ interfaces in the C_bHLH_+C_bHLH_ model is stronger than those in the C_bHLH_+B_bHLH_ and B_bHLH_+B_bHLH_ models due to the stronger negative and positive surface charges respectively at the H2_C_ and H1_C_ helices in the CLOCK protein over that in the BMAL1 protein.

**Table 2:** The occupancies (%) of hydrogen bonds and hydrophobic interactions in the binary C_bHLH_+B_bHLH_, B_bHLH_+B_bHLH_, and C_bHLH_+C_bHLH_ models.

	**Hydrogen bonds**			(Glu94)OE1∙∙∙H-NH1(Arg116)	75
	**C**_**bHLH**_**+B**_**bHLH**_			(Glu94)OE1∙∙∙H-NH2(Arg116)	45
H1_C_-H2_B_	(Arg46)OD1/2-H∙∙∙OD1(Asp110)	37		(Glu94)OE2∙∙∙H-NH2(Arg116)	65
H1_B_-H2_C_	(Asp69)OD1/2∙∙∙H-NH2(Arg84)	78		(Asp110)OD2-H∙∙∙NH1/2(Arg84)	83
	(Arg82)NH2-H∙∙∙OE1/2(Glu94)	88		(Asp110)OD1-H∙∙∙NH1/2(Arg84)	76
	**B**_**bHLH**_**+B**_**bHLH**_			(Arg126)NH1-H∙∙∙O(Leu98)	45
H1_B_-H2_B_	(Glu94)OE2∙∙∙H-NH1(Arg116)	70			
	**Hydrophobic interactions**			(Ile92)CG1∙∙∙CD1(Leu115)	78
	**C**_**bHLH**_**+B**_**bHLH**_			(Ile92)CG1∙∙∙CD2(Leu115)	76
H1_C_-H2_B_	(Leu53)CB∙∙∙CD2(Leu115)	98		(Met122)CB∙∙∙CD2(Leu98)	77
	(Leu53)CB∙∙∙CG(Leu115)	81		**C**_**bHLH**_**+C**_**bHLH**_	
	(Leu57)CD2∙∙∙CD1(Leu115)	96	H1_C_-H2_C_	(Phe50)CB∙∙∙CG (Lys70)	86
	(Leu57)CD1∙∙∙CG(Met122)	80		(Leu53)CB∙∙∙CB(Leu74)	91
	(Met60)CB∙∙∙CB(Met122)	84		(Leu53)CD1∙∙∙CB(Leu74)	83
H1_B_-H2_C_	(Leu74)CB∙∙∙CB(Phe91)	98		(Leu53)CD2∙∙∙CD1(Ile78)	96
	(Ile78)CG2∙∙∙CB(Leu95)	95		(Ser71)CB∙∙∙CD1(Leu53)	82
	(Ile78)CG2∙∙∙CD1(Leu95)	91		(Ile78)CD1∙∙∙CD2(Leu53)	86
	(Ile78)CD1∙∙∙CD1(Leu98)	90		(Ile78)CD1∙∙∙CB(Glu56)	80
H2_C_-H2_B_	(Ser77)CB∙∙∙CE(Met122)	79		(Ile78)CG1∙∙∙CB(Leu57)	88
	**B**_**bHLH**_**+B**_**bHLH**_			(Ile78)CG1∙∙∙CD1(Leu57)	87
H1_B_-H2_B_	(Phe91)CB∙∙∙CB(Leu115)	76			

### 3.2. Stability of the ternary C_bHLH_+B_bHLH_+DNA and B_bHLH_+B_bHLH_+DNA models

#### 3.2.1. Binding free energy calculations and conformational analysis

To address the stability of the ternary C_bHLH_+B_bHLH_+DNA and B_bHLH_+B_bHLH_+DNA models, the binding free energies of protein-protein and protein-DNA were calculated, and are shown in Table 1. The binding free energies of the C_bHLH_+B_bHLH_ and B_bHLH_+B_bHLH_ dimers with DNA are -98.64 and -78.76 kcal·mol^-1^, respectively, which supports that the both dimers can successfully bind to E-box DNA at the H1_C/B_-H1_B/B_ interfaces (see Fig 3B), and that the binding ability of the C_bHLH_+B_bHLH_ heterodimer to DNA is slightly stronger than the B_bHLH_+B_bHLH_ homodimer. However, the binding free energies between their bHLH domains for the two ternary C_bHLH_+B_bHLH_+DNA and B_bHLH_+B_bHLH_+DNA models decrease respectively by 12.42 and 14.22 kcal·mol^-1^ relative to their binary models due to the function of the α-helical forceps (see Fig 3B), that is, the tight gripping of the H1_C/B_/H1_B_ helices respectively located at each of the hetero/homo-bHLH domains to the major groove of DNA would cause the decrease of interactions at the H1_C/B_-H2_B/C_ and H1_B_-H2_B_ interfaces for both C_bHLH_+B_bHLH_+DNA and B_bHLH_+B_bHLH_+DNA models [78].

To explore the interactions of the bHLH domains of the CLOCK and BMAL1 proteins with the DNA molecule, the analyses of hydrogen bonds and hydrophobic interactions for the two ternary systems were performed, and are listed in S2 Table of the Supporting Information. It can be seen that lots of the hydrogen bonds were detected for the C_bHLH_+B_bHLH_+DNA and B_bHLH_+B_bHLH_+DNA models. Especially, for the C_bHLH_+B_bHLH_+DNA model, some binding sites at the residues Arg39, Glu43, Arg47 in the H1_C_ helix, and His77, Glu81, Arg85 in the H1_B_ helix reproduce the experimental results [44]. The hydrophobic interaction between the Ile80 residue of the H1_B_ helix and the T20 base of DNA with the occupation of 85% was detected, and also reproduces the experimental result [44]. The binding sites on the DNA bases of C6, A7, C8, G9, G11, T20, C21, A22, G24 and T25 are consistent with the experimental result of recognition of 7-bp DNA for the heterodimeric bHLH domains of CLOCK and BMAL1 complex in the isothermal titration calorimetry experiment [44]. For the B_bHLH_+B_bHLH_+DNA model, the binding sites of the bases G9, T10, G11, G24, T25 and G26 reveal that the bHLH domains of BMAL1 homodimer can recognize 6-bp DNA, which is also consistent with some experimental results [79, 80]. Because the DNA in this work is the palindromic canonical form of E-box, each H1_B_ helix in the homomeric B_bHLH_+B_bHLH_+DNA model can symmetrically bind to the GTG bases of the DNA molecule near by the 3’ terminal of each strand of DNA as a diagonal binding mode. However, the H1_C_ and H1_B_ helices belonged to the different proteins in the heteromeric C_bHLH_+B_bHLH_+DNA model bind asymmetrically to the GTGCACT bases located at the center of the DNA molecule, as a rectangular binding mode, with the stronger affinity over that in the homomeric B_bHLH_+B_bHLH_+DNA model. Moreover, the conformational analysis reveals there are more binding sites located at the H1_C_-H1_B_ interface and the DNA molecule in the heteromeric C_bHLH_+B_bHLH_+DNA model over that in the homomeric B_bHLH_+B_bHLH_+DNA model due to the big DNA-bound interhelical angle between the H1_C_ and H1_B_ helices in the heteromeric C_bHLH_+B_bHLH_+DNA model, which will be discussed as follows. It can be revealed by the electrostatic surface potential analysis shown in Fig 4 that the strong positive surface charges of the H1_C_ and H1_B_ helices might electrostatically favor to bind to the negative charged DNA molecule. Moreover, it can be seen from Table 2 and S2 Table that the numbers of total hydrogen bonds and total hydrophobic interactions at the H1_C/B_-H2_B/C_ interfaces decrease from 203 and 813 to 147 and 530, respectively, during their simulation times, in the C_bHLH_+B_bHLH_+DNA model compared to that in the C_bHLH_+B_bHLH_ model (The corresponding calculation details are given in S3 Text of the Supporting Information). And the original hydrophobic interaction at the H2_C_-H2_B_ interface in the C_bHLH_+B_bHLH_ model disappears in the C_bHLH_+B_bHLH_+DNA model. These results support the decrease of binding free energy of protein-protein.

Moreover, the H1_C/B_ helices in CLOCK and BMAL1 binding to the major groove of DNA cause the DNA conformation disturbance. The DNA groove parameters along with the DNA base pairs of C6A7C8G9T10G11A12 that are involved in the rectangular and diagonal binding modes, were analyzed for the C_bHLH_+B_bHLH_+DNA, B_bHLH_+B_bHLH_+DNA and B-DNA models, and are shown in S3 Fig of the Supporting Information. It can be seen that the major/minor groove widths and depths of DNA are changed along with the base pairs with respect to B-DNA. Especially, the increases of major groove depth and minor groove width of DNA are along with the decreases of major groove width and minor groove depth, respectively, in the C_bHLH_+B_bHLH_+DNA and B_bHLH_+B_bHLH_+DNA models, and vice versa. The average major groove depths calculated from the bound base pairs in the C_bHLH_+B_bHLH_+DNA and B_bHLH_+B_bHLH_+DNA models are increased by 22% and 29% compared to that in the normal B-DNA model, respectively. Their corresponding average minor depths become shallow by 11% and 13% for the C_bHLH_+B_bHLH_+DNA and B_bHLH_+B_bHLH_+DNA models, respectively. As expected, the conformation disturbance of the DNA molecule caused by the binding of the bHLH domains of CLOCK and BMAL1 further supports the strong binding affinity between the proteins and DNA.

#### 3.2.2. Dynamic fluctuation and correlation for the binary C_bHLH_+B_bHLH_ and ternary C_bHLH_+B_bHLH_+DNA models

**Fig 5:**
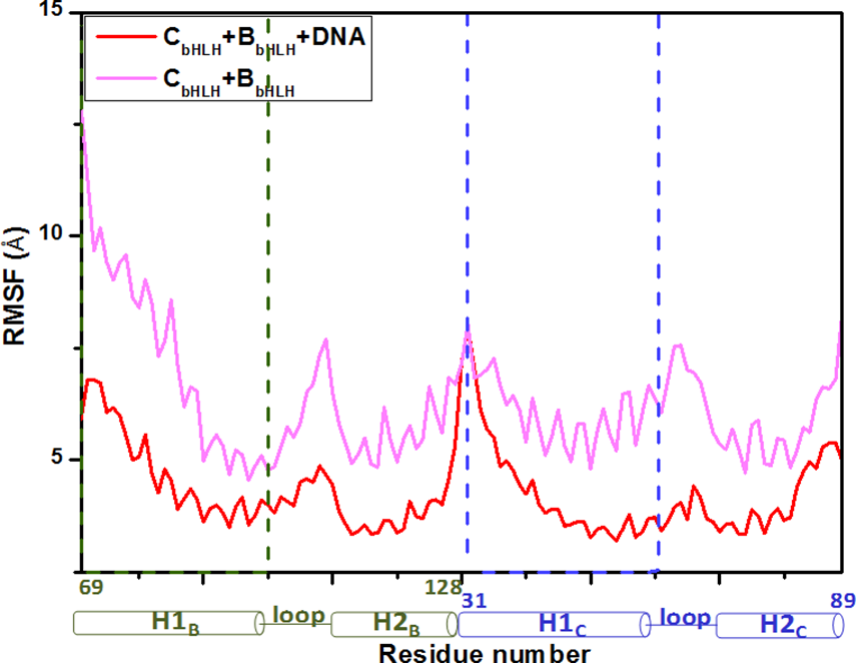
The fluctuations of residues and bases in the C_bHLH_+B_bHLH_ and C_bHLH_+B_bHLH_+DNA models. The fluctuations of residues and bases in the C_bHLH_+B_bHLH_ (light magenta) and C_bHLH_+B_bHLH_+DNA (red) models.

To address the interactions of the bHLH domains with the DNA molecule and the conformational changes for the C_bHLH_+B_bHLH_ and C_bHLH_+B_bHLH_+DNA models, the dynamics of every residue/base were determined and interpreted by residue/base fluctuations and correlations. The corresponding RMSF values were analyzed (see Fig 5). It can be observed that the strong interactions between the H1_C/B_ helix and the DNA molecule make the H1_C/B_ helix more stable with the RMSF decrease of 1–6 Å in the ternary C_bHLH_+B_bHLH_+DNA model than that in the binary C_bHLH_+B_bHLH_ model due to the DNA binding sites located at the H1_C/B_ helix in CLOCK or BMAL1 discussed above. These results further support the interaction analysis.

**Fig 6:**
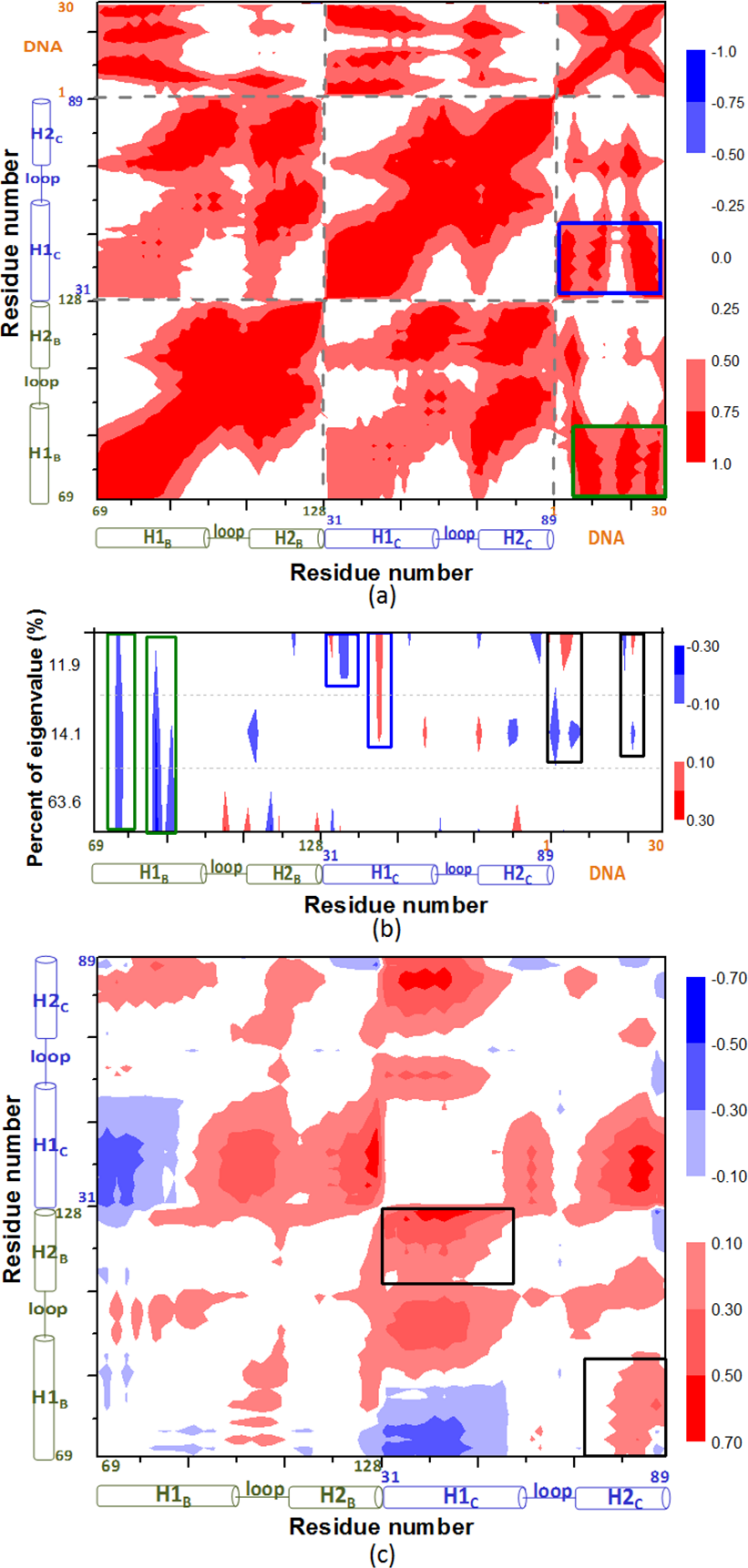
Motion correlations, eigenvector map and differences of the protein motion correlations of the C_bHLH_+B_bHLH_ and C_bHLH_+B_bHLH_+DNA models. (a) Motion correlations of the C_bHLH_+B_bHLH_+DNA model with the key subregions of CLOCK and BMAL1 squared in blue and green, respectively. (b) Eigenvector map of the corresponding matrix with the first three larger eigenvalues in the C_bHLH_+B_bHLH_+DNA model. The blue, green and black squares in (b) presented the same meaning in (a). (c) The differences of the protein motion correlations from the C_bHLH_+B_bHLH_ to C_bHLH_+B_bHLH_+DNA models with the specific subregions squared in black.

To further explore the affinity of the bHLH domains of the CLOCK and BMAL1 proteins with DNA, the motion correlations for each Cα atom of residue and each Phosphorus atom of base in the C_bHLH_+B_bHLH_+DNA model from its trajectory were analyzed, and are shown in Fig 6A. It is shown that the motion correlations between the residues/bases range from highly anti-correlated (blue) to highly correlated (red). As illustrated in Fig 6A the motions of the residues in the H1_C_ and H1_B_ helices significantly correlate with that of the bases in the DNA molecule (represented by the blue and green squares, respectively, in Fig 6A), which supports the strong interactions between the H1_C/B_ helix and DNA in the C_bHLH_+B_bHLH_+DNA model. Moreover, to yield the most significant correlated motions, the three eigenvectors of the correlation matrix with the first three larger eigenvalues (>90%) were used to find out the dominant correlated regions for the C_bHLH_+B_bHLH_+DNA model, and are shown in Fig 6B. It can be found that the dominant correlated regions mainly occur at the H1_C_ (blue squares) and H1_B_ helices (green squares), and the central DNA bases (black squares) in the C_bHLH_+B_bHLH_+DNA model, which supports the corresponding cross-correlation analysis in Fig 6A. To compare the interactional variation between the bHLH domains of the CLOCK and BMAL1 proteins in the ternary and binary models, the differences of motion correlations of the residues in the ternary C_bHLH_+B_bHLH_+DNA model from that in the binary C_bHLH_+B_bHLH_ model were conducted, and are shown in Fig 6C. The negative and positive values in Fig 6C mean the decrease and increase magnitudes of correlation coefficients of residues from the binary C_bHLH_+B_bHLH_ model to the ternary C_bHLH_+B_bHLH_+DNA model, respectively. It can be seen that the motion correlations of the H1_C_ helix with the H2_B_ helix, and the H1_B_ helix with the H2_C_ helix at the H1_C/B_-H2_B/C_ interfaces in the ternary C_bHLH_+B_bHLH_+DNA model are decreased, compared to that in the binary C_bHLH_+B_bHLH_ model (represented by the black squares in Fig 6C) due to the additional interaction between the H1_C/B_ helices and DNA with the function of the α-helical forceps. These results support the binding free energy and interaction analyses discussed above. The similar decreasing of motion correlations between the proteins was also found from the homomeric B_bHLH_+B_bHLH_ model to B_bHLH_+B_bHLH_+DNA model.

#### 3.2.3. Phosphorylation analysis for the ternary C_bHLH_+B_Phos_+DNA and B_Phos_+B_Phos_+DNA models

**Fig 7:**
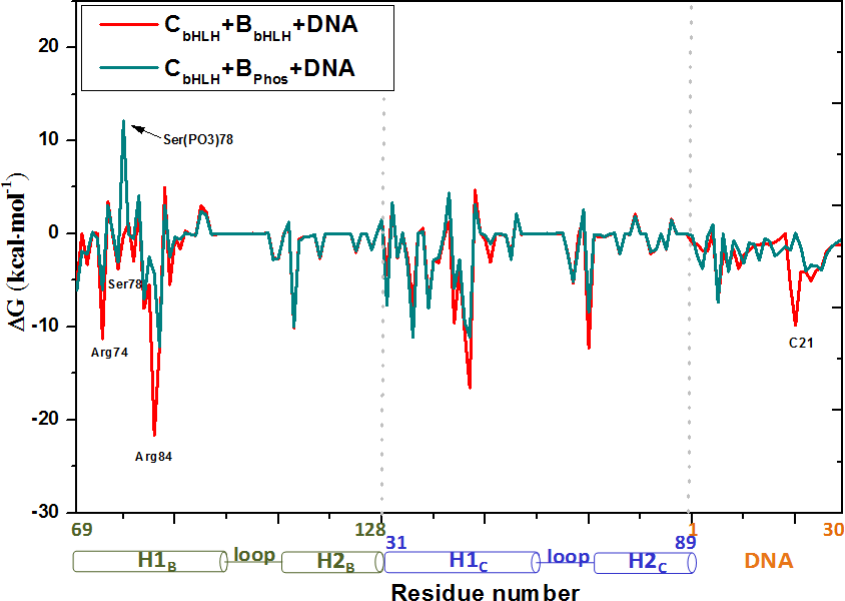
Energy decompositions of the C_bHLH_+B_bHLH_+DNA and C_bHLH_+B_Phos_+DNA models. MM-PBSA energy decompositions (kcal·mol^-1^) into the residues of the bHLH domains and the bases of the DNA molecule for the C_bHLH_+B_bHLH_+DNA (red) and C_bHLH_+B_Phos_+DNA (sky blue) models.

**Fig 8:**
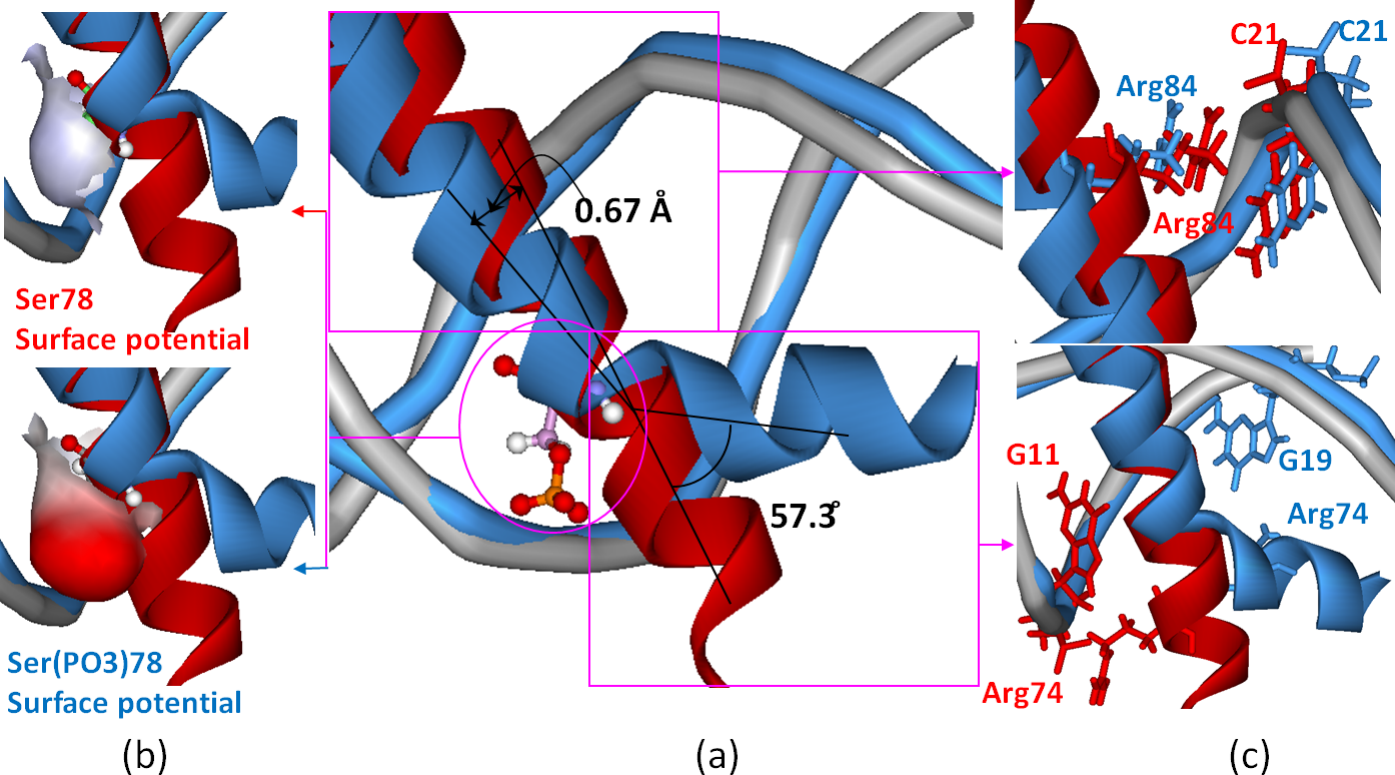
Conformation differences of the C_bHLH_+B_bHLH_+DNA and C_bHLH_+B_Phos_+DNA models. (a) The conformational difference of the H1_B_ helix, (b) the different electrostatic surface potentials of the residue of Ser78/Ser(PO3)78, and (c) the different positions of the key residues in the C_bHLH_+B_bHLH_+DNA (red) and C_bHLH_+B_Phos_+DNA (sky blue) models.

The phospho-mimicking experiment showed that the single mutation of Ser78Glu in the H1_B_ helix almost abolishes the binding of the bHLH domains to DNA in the CLOCK/BMAL1-DNA complex [44]. To further explore the phosphorylation mechanism, we performed the MD simulations for the phosphorylated C_bHLH_+B_Phos_+DNA and B_Phos_+B_Phos_+DNA models in which Ser78 located at each H1_B_ helix was phosphorylated to Ser(PO3)78 based on the previous works [60, 61]. The corresponding binding free energies and hydrogen bond occupancies were calculated, and are shown in S3 and S4 Tables of the Supporting Information, respectively. It can be seen that the binding free energies between the bHLH domains and DNA in the phosphorylated C_bHLH_+B_Phos_+DNA and B_Phos_+B_Phos_+DNA models decrease by 43.59 and 37.73 kcal·mol^-1^, respectively, compared to that in the non-phosphorylated C_bHLH_+B_bHLH_+DNA and B_bHLH_+B_bHLH_+DNA models (see Table 1 and S3 Table). To further understand the phosphorylation mechanism of Ser(PO3)78 in the H1_B_ helix, the decompositions of the corresponding free energy into the residues and bases for the C_bHLH_+B_bHLH_+DNA and C_bHLH_+B_Phos_+DNA models were carried out, and are shown in Fig 7. The decompositions of binding free energy would provide more quantitative information of energy contribution for each residue or base. It can be seen that the distinct differences of binding free energy decompositions in the two models occur mainly at the residues Arg74, Ser78, Arg84 in the H1_B_ helix and the base C21 of DNA in the phosphorylated C_bHLH_+B_Phos_+DNA model with the decrease magnitudes of binding free energies by 5.28, 12.32, 17.36 and 9.91 kcal·mol^-1^, respectively, compared with those in the non-phosphorylated C_bHLH_+B_bHLH_+DNA model. To investigate the structural variation of the ternary bHLH domains of the CLOCK and BMAL1 proteins with DNA induced by the Ser78 phosphorylation, the superimposed average structures and the electrostatic surface potentials of the Ser78/Ser(PO3)78 residues for the phosphorylated C_bHLH_+B_Phos_+DNA and non-phosphorylated C_bHLH_+B_bHLH_+DNA models extracted from their simulations are shown in Fig 8A–8C. It can be seen that the relatively big size and the negative surface charges of the Ser(PO3)78 residue inhibit the binding of the bHLH domains of the CLOCK and BMAL1 proteins to the DNA molecule, and cause the shifting of the H1_B_ helix by 0.67 Å far from DNA and the tail tilting of the H1_B_ helix by 57.3° in the phosphorylated C_bHLH_+B_Phos_+DNA model. Such shifting and tail tilting result in the abolishing of binding of Arg84 in the H1_B_ helix to the base C21 of DNA with the decrease of the hydrogen bond occupancy by 100%, and the decrease of electrostatic interaction of Arg74 in the H1_B_ helix with the base G11 or G19 of DNA (see S4 Table and Fig 8), which further illustrate the energy-decreased contributions of the key residues discussed in the decomposition analysis of binding free energy.

### 3.3. Analysis of binding free energies of protein-protein and protein-DNA for PAS domains of the CLOCK and BMAL1 proteins

To address the influence of the PAS domains on the bindings of bHLH-bHLH and bHLH-DNA, the binding free energy calculations for protein-protein in the C_bHLH_+B_bHLH_+PAS model, and for protein-protein and protein-DNA in the C_bHLH_+B_bHLH_+PAS+DNA model were carried out, and are given in S3 Table of the Supporting Information. It can be seen that the binding free energy of -82.99 kcal·mol^-1^ for protein-DNA in the C_bHLH_+B_bHLH_+PAS+DNA model is almost equal to that of -98.64 kcal·mol^-1^ in the C_bHLH_+B_bHLH_+DNA model. So the PAS domains affect insignificantly the affinity of the CLOCK and BMAL1 proteins with the DNA molecule. However, the PAS domains significantly enhance the affinity between the CLOCK and BMAL1 proteins with the binding free energies of -352.81 and -12.55 kcal·mol^-1^, respectively, for the C_bHLH_+B_bHLH_+PAS and C_bHLH_+B_bHLH_ models. Similarly, the binding free energy between the bHLH-PAS domains in the CLOCK and BMAL1 proteins for the C_bHLH_+B_bHLH_+PAS+DNA model decreases by 23.02 kcal·mol^-1^ relative to its binary C_bHLH_+B_bHLH_+PAS model due to the DNA binding, which can be also explained by the function of the α-helical forceps of the bHLH domains (see Fig 3C).

## 4. Discussion

### 4.1. Analyses of residue charges and helical distances at the H1-H2 interfaces

**Fig 9:**
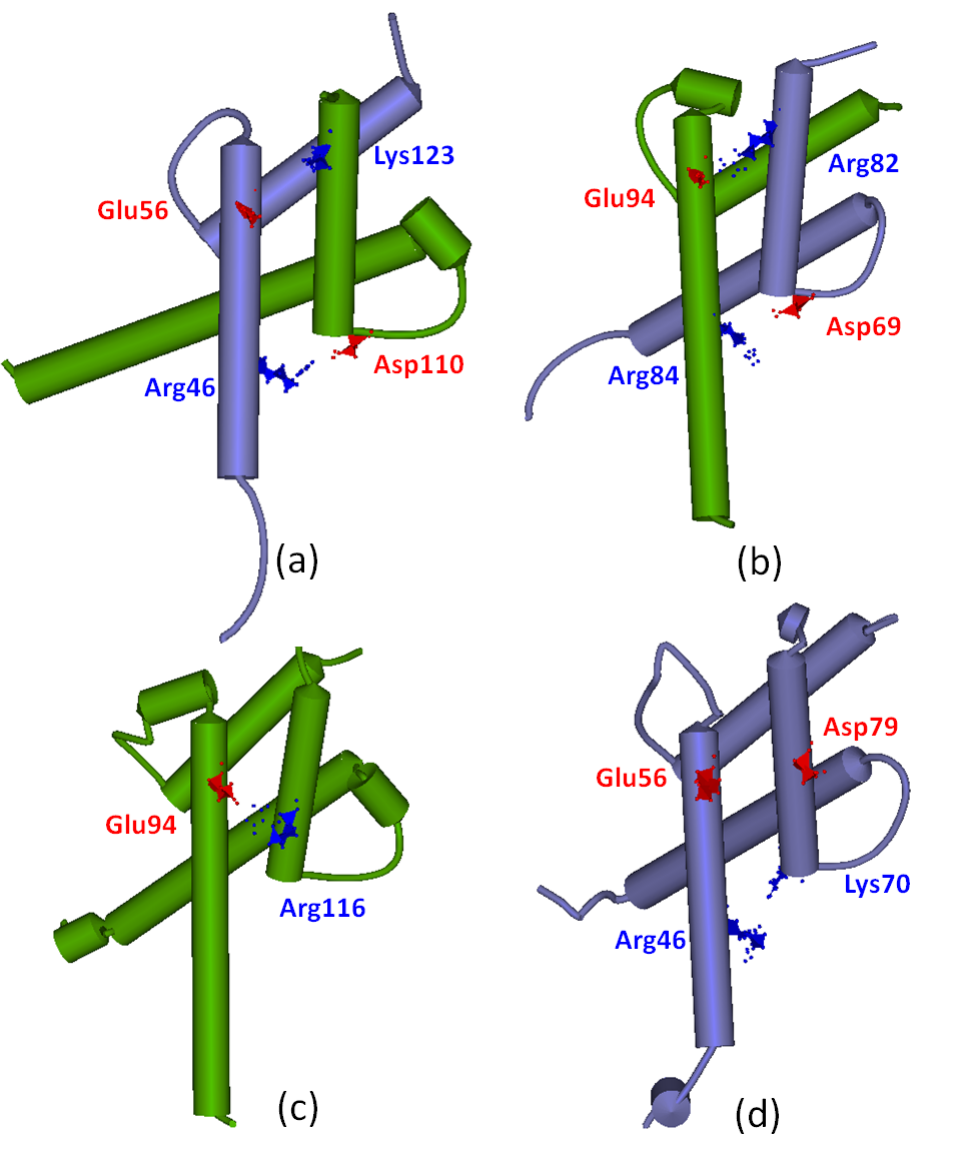
Charged residues in the binary models. Some positive-charged residues (blue polyhedron) and the negative-charged residues (red polyhedron) at the H1-H2 interfaces in the C_bHLH_+B_bHLH_ model ((a) and (b)), the B_bHLH_+B_bHLH_ model (c), and the C_bHLH_+C_bHLH_ model (d)

**Fig 10:**
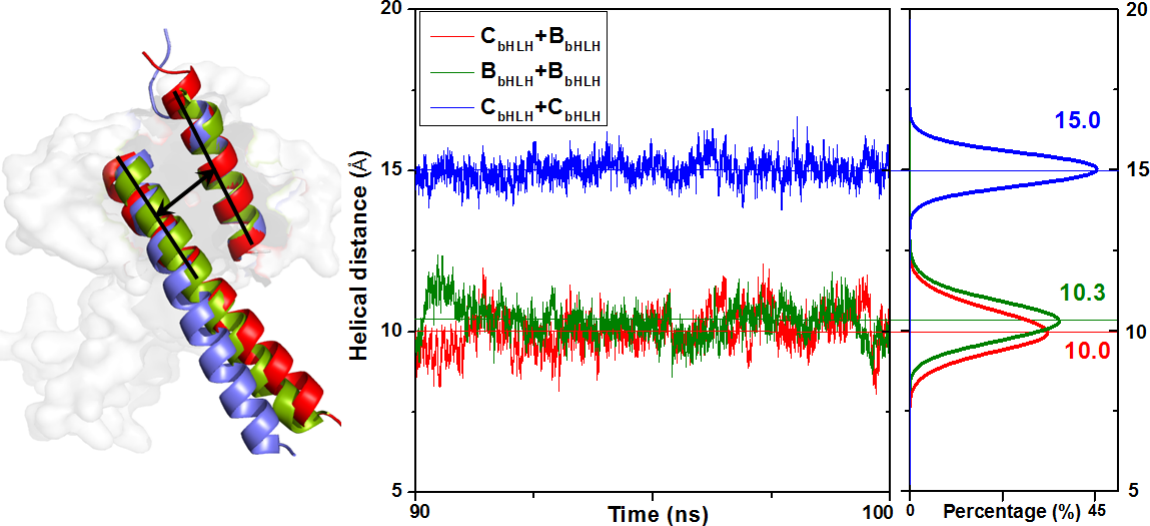
The helical distances in the binary models. The helical distances (Å) between the H1 and H2 helices in the C_bHLH_+B_bHLH_ (red), B_bHLH_+B_bHLH_ (green), and C_bHLH_+C_bHLH_ (blue) models.

The residue charge analysis at the H1_C/B_-H2_B/C_ and H1_B/C_-H2_B/C_ interfaces was applied to further investigate the stability of three binary C_bHLH_+B_bHLH_, B_bHLH_+B_bHLH_ and C_bHLH_+C_bHLH_ models, and is shown in Fig 9A–9D. It can be seen that the residue charges at the H1_C/B_-H2_B/C_ and H1_B/C_-H2_B/C_ interfaces favor the stability of the C_bHLH_+B_bHLH_ and B_bHLH_+B_bHLH_ dimers over that of the C_bHLH_+C_bHLH_ one. Namely, for the H1_C_-H2_B_ interface of the C_bHLH_+B_bHLH_ model in Fig 9A, the positive charged Arg46 and negative charged Glu56 residues in CLOCK can considerably attract the adjacent negative charged Asp110 and positive charged Lys123 residues in BMAL1, respectively. The similar attractions between the negative charged Asp69 residue of CLOCK and the positive charged Arg84 residue of BMAL1, and between the positive charged Arg82 residue of CLOCK and the negative charged Glu94 residue of BMAL1 could occur at the H1_B_-H2_C_ interface (see Fig 9B). The calculated hydrogen bond between Arg82 of CLOCK and Glu94 of BMAL1 at the H1_B_-H2_C_ interface with the simulation occupancy of 88% also supports such charge analysis. For the H1_B_-H2_B_ interfaces of the B_bHLH_+B_bHLH_ model, the negative charged Glu94 residue attracts the adjacent positive charged Arg116 residue in each BMAL1 (see Fig 9C). Similarly, the hydrogen bonds between them support the charge analysis. However, for the H1_C_-H2_C_ interfaces of the C_bHLH_+C_bHLH_ model, the negative charged Glu56 residue and the positive charged Arg46 residue in one CLOCK repulse the negative charged Asp79 residue and the positive charged Lys70 residues in other CLOCK, respectively (see Fig 9D). As expected, none of the hydrogen bonds was found in these residues. Such residue charge analysis also supports the results of electrostatic surface charges discussed above (see Fig 4). Moreover, the average helical distances between the H1_C_ and H2_B_ helices, and between the H1_B/C_ and H2_B/C_ helices for the C_bHLH_+B_bHLH_, B_bHLH_+B_bHLH_ and C_bHLH_+C_bHLH_ models over the simulation times were calculated, and are shown in Fig 10. It can be seen that the calculated helical distances of 10.0 and 10.3 Å between the H1_C_ and H2_B_ helices, and between the H1_B_ and H2_B_ helices for the C_bHLH_+B_bHLH_ and B_bHLH_+B_bHLH_ models are all smaller than that of 15.0 Å for the C_bHLH_+C_bHLH_ model, which supports the stronger interactions at the H1_C_-H2_B_ and H1_B_-H2_B_ interfaces of the C_bHLH_+B_bHLH_ and B_bHLH_+B_bHLH_ models over that of the C_bHLH_+C_bHLH_ model. That is, both hydrogen bonds and hydrophobic interactions at the H1_C/B_-H2_B/C_ and H1_B_-H2_B_ interfaces were found for the C_bHLH_+B_bHLH_ and B_bHLH_+B_bHLH_ models. However, only hydrophobic interactions at the H1_C_-H2_C_ interfaces were found for the C_bHLH_+C_bHLH_ model.

### 4.2. Comparison of binding mode of the C_bHLH_+B_bHLH_+DNA model with that of the B_bHLH_+B_bHLH_+DNA model

**Fig 11:**
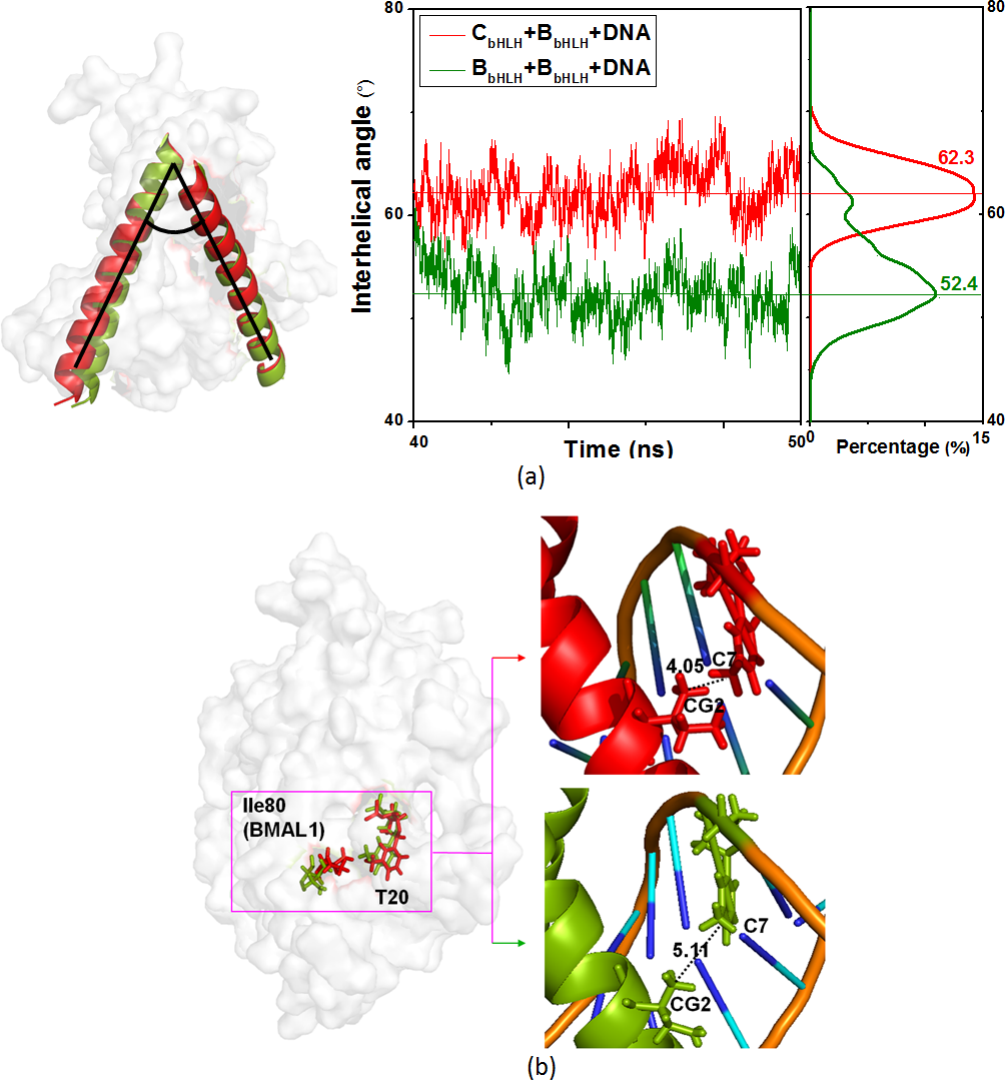
The interhelical angles and the distances of the C_bHLH_+B_bHLH_+DNA and B_bHLH_+B_bHLH_+DNA models. (a) The interhelical angles (°) between the H1 helices, and (b) the distances (Å) between the C atoms for Ile80 in the H1 helix of BMAL1 and T20 in DNA in the C_bHLH_+B_bHLH_+DNA (red) and B_bHLH_+B_bHLH_+DNA (green) models.

Due to the palindromic canonical form of the current E-box DNA molecule, the bindings of the H1_C/B_ helices in the CLOCK and BMAL1 proteins to DNA form the rectangular and diagonal binding modes for the heteromeric C_bHLH_+B_bHLH_+DNA model and the homomeric B_bHLH_+B_bHLH_+DNA model, respectively. The numbers of the main bound residues and bases (Arg36, Arg39, Asp40, Glu43, Arg46, Arg47 in the H1_C_ helix, and Arg74, His77, Ile80, Glu81, Arg85 in the H1_B_ helix, and C6, A7, C8, G9, G11, T20, C21, A22, G24, T25) in the rectangular binding mode for the C_bHLH_+B_bHLH_+DNA model are more than those (Arg74, His77, Glu81, Arg85 in each of the H1_B_ helices, and C6, G9, T10, G11, C21, G24, T25, G26) in the diagonal binding mode for the B_bHLH_+B_bHLH_+DNA model, resulting in the strong interactions of the proteins and DNA for the C_bHLH_+B_bHLH_+DNA model. Moreover, it can be seen from the electrostatic surface potentials shown in Fig 4 that the positive surface charges at the H1_C_ helix are larger than that at the H1_B_ helix, which causes more binding sites between the positive charged H1_C/B_ helices and the negative charged DNA molecule in the rectangular binding mode for the C_bHLH_+B_bHLH_+DNA model than that in the diagonal binding mode for the B_bHLH_+B_bHLH_+DNA model. The average interhelical angles between the H1_C_ and H1_B_ helices, and between the two H1_B_ helices, and the distances between the C atom of Ile80 residue in BMAL1 and the C atom of the T20 base in DNA for the C_bHLH_+B_bHLH_+DNA and B_bHLH_+B_bHLH_+DNA models over the simulation times were investigated, and are shown in Fig 11A and 11B. It can be seen that the larger interhelical angle of 62.3° in the C_bHLH_+B_bHLH_+DNA model over that of 52.4° in the B_bHLH_+B_bHLH_+DNA model can contribute to the large binding surface to the DNA molecule at the H1_C_-H1_B_ interface for the C_bHLH_+B_bHLH_+DNA model. Additionally, it can be seen from Fig 11B that the average distances of 4.05 and 5.11 Å between the C atom of Ile80 residue in BMAL1 and the C atom of T20 base in DNA for the C_bHLH_+B_bHLH_+DNA and B_bHLH_+B_bHLH_+DNA models, respectively, also reveal the stronger interaction for the rectangular binding mode in the C_bHLH_+B_bHLH_+DNA model over that for the diagonal binding mode in the B_bHLH_+B_bHLH_+DNA model, which also supports their hydrophobic interaction measured by the experiment [44]. For the corresponding RMSF analysis that is shown in S4 Fig of the Supporting Information, the smaller RMSF values of the bHLH domains and the DNA molecule in the C_bHLH_+B_bHLH_+DNA model than that in the B_bHLH_+B_bHLH_+DNA model also reveals that the heteromeric C_bHLH_+B_bHLH_+DNA model with the rectangular binding mode is more stable than the homomeric B_bHLH_+B_bHLH_+DNA model with the diagonal binding mode due to the more favorable DNA binding in the C_bHLH_+B_bHLH_+DNA model. As expected, the stable rectangular binding mode of the C_bHLH_+B_bHLH_+DNA model presents more hydrogen bonds by 11% between the H1_C/B_ helices and DNA than that for the diagonal binding mode of the B_bHLH_+B_bHLH_+DNA model.

### 4.3. Influence of the PAS domains on binding features

To address the significant effect of the PAS domains on the binding features between the bHLH-PAS domains of CLOCK and BMAL1 in the C_bHLH_+B_bHLH_+PAS+DNA model, the analyses of hydrogen bonds and electrostatic surface potentials of the bHLH-PAS domains were carried out, and are shown in S5 Table and S5 Fig of the Supporting Information, respectively. The number of the total hydrogen bonds at the PAS_C_-PAS_B_ interface is 1694 from the trajectory in the C_bHLH_+B_bHLH_+PAS+DNA model, which favors its stability. The electrostatic surface charges of the PAS domains in CLOCK and BMAL1 are mainly negative and positive, respectively, which also favors the combination of the bHLH-PAS domain in CLOCK with that in BMAL1 (see S5 Fig). For example, the simulation occupation times of 97% and 93% of the hydrogen bonds between the negative charged Asp119 residue in CLOCK and the positive charged Arg319 residue in BMAL1, and between the negative charged Asp311 residue in CLOCK and the positive charged Arg343 residue in BMAL1, respectively, were found at the PAS_C_-PAS_B_ interface in the C_bHLH_+B_bHLH_+PAS+DNA model (see S5 Table). Furthermore, it can be seen from the RMSF values showed in S6 Fig of the Supporting Information that the RMSF values for the residues of the bHLH domains and the bases of DNA in the C_bHLH_+B_bHLH_+PAS+DNA model are smaller than that in the C_bHLH_+B_bHLH_+DNA model, which also supports the stability of the C_bHLH_+B_bHLH_+PAS+DNA model. For the influence of the PAS domains on the DNA binding, the number of the total hydrogen bonds of 1559 from the trajectory at the H1_C/B_-DNA interfaces in the C_bHLH_+B_bHLH_+PAS+DNA model almost equivalent to that of 1631 in the C_bHLH_+B_bHLH_+DNA model predicts that the PAS domains affect insignificantly the affinity of the CLOCK and BMAL1 proteins with the DNA molecule (see S2 and S5 Tables). The reason might come from the flexible and long loop linkers located at the middle of the PAS and bHLH domains for the CLOCK and BMAL1 proteins (see Fig 3C).

## 5. Conclusions

Molecular dynamics simulations, free energy calculations and DNA dynamics analysis for a series of the constructed models, the bHLH and bHLH-PAS domains of the CLOCK and BMAL1 proteins with and without the DNA molecule, have been performed to address hetero-/homo-dimerization, DNA combination, and phosphorylation and PAS domains influences for the CLOCK and BMAL1 proteins. The dimer of the bHLH domains of CLOCK and BMAL1 presents a four-helical cross-bundle structure with the intertwined mode. The results demonstrate that the bHLH domains of CLOCK and BMAL1 can form a heterodimer of the bHLH domains of CLOCK and BMAL1, and a homodimer of the bHLH domains of BMAL1 with the binding free energies of -12.55 and -14.11 kcal·mol^-1^, respectively. Both heterodimer and homodimer of the bHLH domains could bind to E-box DNA at the H1-H1 interfaces with the binding free energies of -98.64 and -78.76 kcal·mol^-1^, respectively. The bindings of the H1 helices to DNA in the heterodimer and homodimer of the bHLH domains of the CLOCK and BMAL1 proteins show the rectangular and diagonal binding modes, respectively, due to the palindromic canonical form of E-box DNA. However, two binding modes cause the insignificant difference for the conformation disturbance of the DNA molecule. Due to the function of the α-helical forceps in the hetero- and homo-bHLH dimers, the tight gripping of the H1 helices to the major groove of DNA would cause the decrease of interactions at the H1-H2 interfaces in the CLOCK and BMAL1 proteins. The results also show that the relatively big size and the negative surface charges of the Ser(PO3)78 residue in the phosphorylated heterodimer and homodimer of the CLOCK and BMAL1 proteins inhibit the binding of the bHLH domains of the CLOCK and BMAL1 proteins to the DNA molecule. As expected, the additional PAS domains in the CLOCK and BMAL1 proteins affect insignificantly the affinity of the CLOCK and BMAL1 proteins with the DNA molecule due to the flexible and long loop linkers located at the middle of the PAS and bHLH domains, but significantly enhance the affinity between the CLOCK and BMAL1 proteins. These results provide the better understanding for the interactions of the hetero-/homo-bHLH domains and the mechanism of circadian rhythms regulated by the CLOCK and BMAL1 proteins binding to DNA.

## Supporting Information

S1 Fig RMSD values and the superposition of the average structures of the C_bHLH_+B_bHLH_ model for three independent MD simulations. (a) RMSD values of all heavy atoms with respect to the starting structure for three independent MD simulations of the C_bHLH_+B_bHLH_ model, and (b) the superposition of the average structures of the C_bHLH_+B_bHLH_ model extracted from three independent MD simulations.
(TIF)

S2 Fig RMSD values of the C_bHLH_+B_bHLH_+DNA, B_bHLH_+B_bHLH_+DNA, C_bHLH_+B_bHLH_+PAS and C_bHLH_+B_bHLH_+PAS+DNA models. RMSD values of all heavy atoms with respect to the experimental crystal structure and the corresponding starting structures for the MD simulations of (a) the C_bHLH_+B_bHLH_+DNA model, (b) the B_bHLH_+B_bHLH_+DNA model, and (c) the C_bHLH_+B_bHLH_+PAS and C_bHLH_+B_bHLH_+PAS+DNA models.
(TIF)

S3 Fig Groove widths and depths of the B-DNA, C_bHLH_+B_bHLH_+DNA and B_bHLH_+B_bHLH_+DNA models. Groove widths and depths of the B-DNA (black line with square), C_bHLH_+B_bHLH_+DNA (red line with circle) and B_bHLH_+B_bHLH_+DNA (green line with up-triangle) models. (a) Major groove widths, (b) major groove depths, (c) minor groove widths and (d) minor groove depths for the time-averaged structures of DNA conformations.
(TIF)

S4 Fig The fluctuations of residues and bases in the C_bHLH_+B_bHLH_+DNA and B_bHLH_+B_bHLH_+DNA models. The fluctuations of residues and bases in the C_bHLH_+B_bHLH_+DNA (red) and B_bHLH_+B_bHLH_+DNA (green) models.
(TIF)

S5 Fig The electrostatic surface potentials of the bHLH-PAS domains in the C_bHLH_+B_bHLH_+PAS+DNA model. The electrostatic surface potentials for the bHLH-PAS domains of the CLOCK and BMAL1 proteins in the C_bHLH_+B_bHLH_+PAS+DNA model.
(TIF)

S6 Fig The fluctuations of residues and bases in the C_bHLH_+B_bHLH_+DNA and C_bHLH_+B_bHLH_+PAS+DNA models. The fluctuations of residues and bases in the C_bHLH_+B_bHLH_+DNA (red) and C_bHLH_+B_bHLH_+PAS+DNA (violet) models.
(TIF)

S1 Table Components of MM-PBSA binding free energies (kcal·mol^-1^) calculated from three independent MD simulations, and the average ΔG_binding_ with the error range for the C_bHLH_+B_bHLH_ model. (PDF)

S2 Table The occupancies (%) of hydrogen bonds and hydrophobic interactions of protein-DNA and protein-protein in the C_bHLH_+B_bHLH_+DNA and B_bHLH_+B_bHLH_+DNA models. (PDF)

S3 Table Components of MM-PBSA free energies (kcal·mol^-1^) for the C_bHLH_+B_Phos_+DNA, B_Phos_+B_Phos_+DNA, C_bHLH_+B_bHLH_+PAS and C_bHLH_+B_bHLH_+PAS+DNA models. (PDF)

S4 Table The occupancies (%) of hydrogen bonds of protein-DNA in the C_bHLH_+B_Phos_+DNA and B_Phos_+B_Phos_+DNA models. (PDF)

S5 Table The occupancies (%) of hydrogen bonds of protein-DNA and protein-protein in the C_bHLH_+B_bHLH_+PAS+DNA model. (PDF)

S1 Text Molecular dynamics simulation protocols. (PDF)

S2 Text MM-PBSA calculation for free energy. (PDF)

S3 Text Analyses of fluctuation, correlation, interaction, interhelical angle/distance, and DNA groove parameter. (PDF)
