# Supplementary figures and images for: Investigations of the CLOCK and BMAL1 Proteins Binding to DNA: A Molecular Dynamics Simulation Study
Source: PLoS One. 2016 May 6;11(5):e0155105. doi: 10.1371/journal.pone.0155105 (PMC4859532; doi:10.1371/journal.pone.0155105)

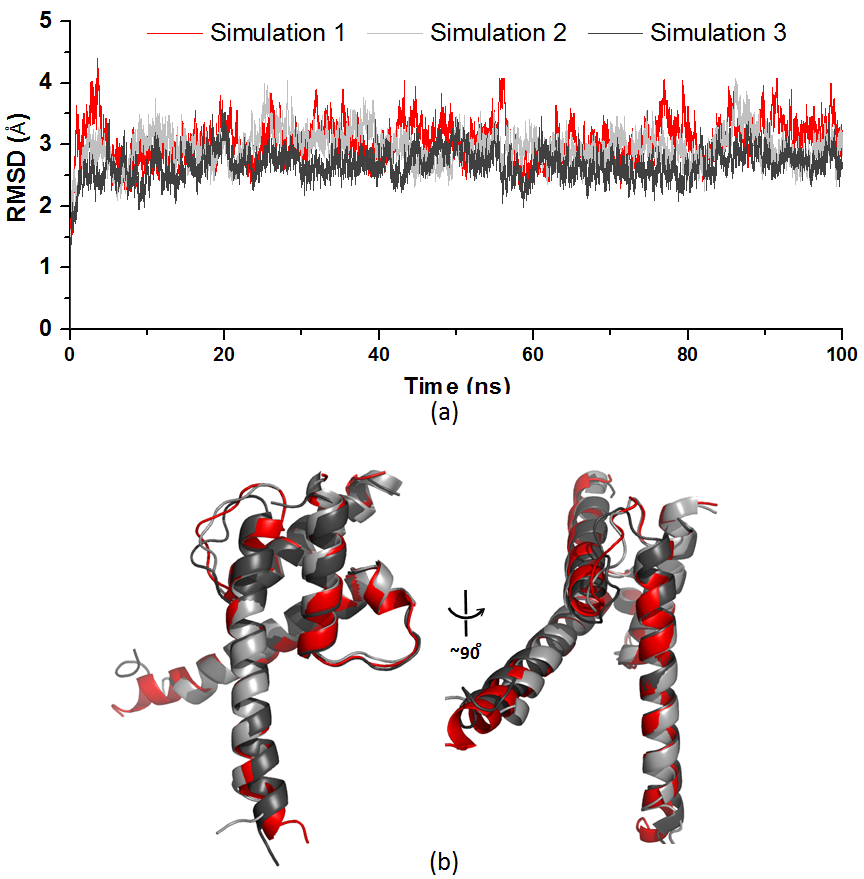

Supplement: S1 Fig — (a) RMSD values of all heavy atoms with respect to the starting structure for three independent MD simulations of the CbHLH+BbHLH model, and (b) the superposition of the average structures of the CbHLH+BbHLH model extracted from three independent MD simulations. (TIF) [file pone.0155105.s001.tif]

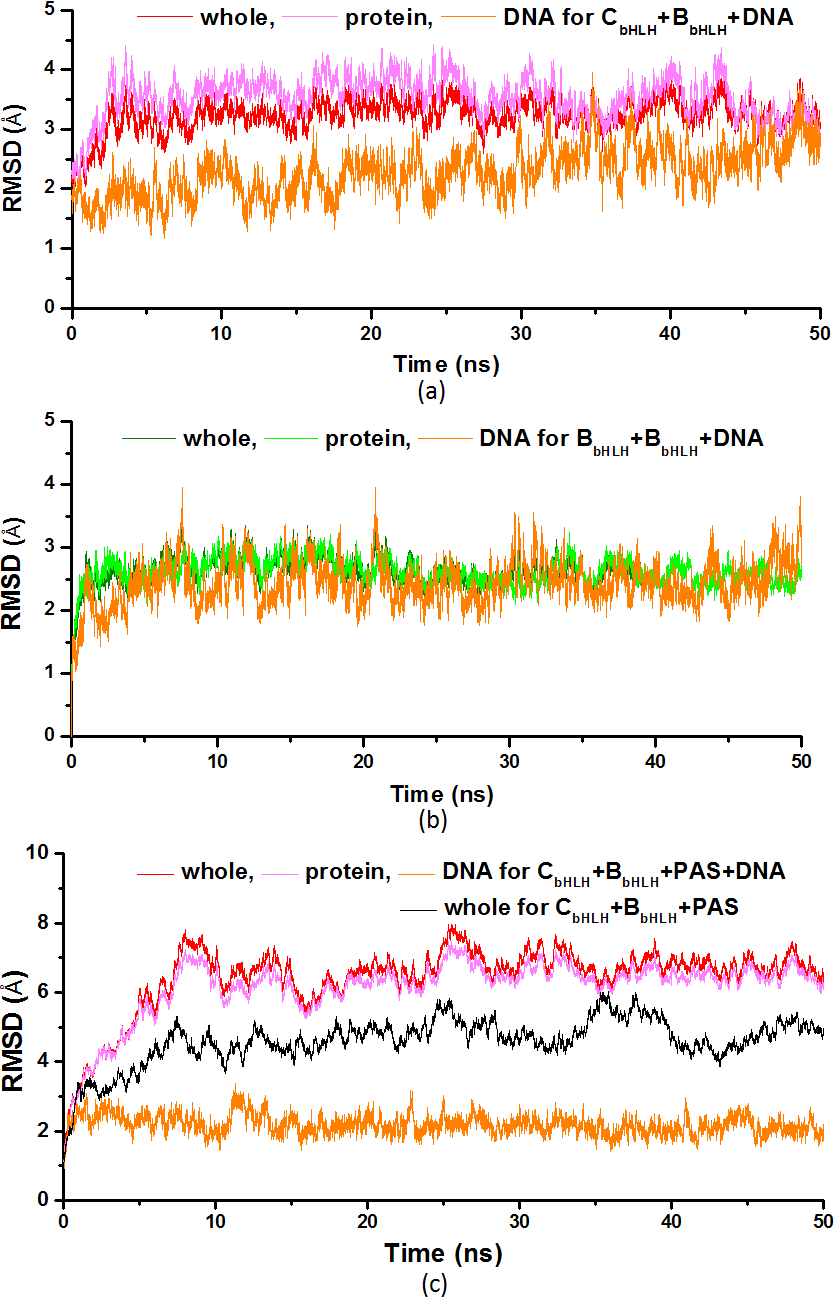

Supplement: S2 Fig — RMSD values of all heavy atoms with respect to the experimental crystal structure and the corresponding starting structures for the MD simulations of (a) the CbHLH+BbHLH+DNA model, (b) the BbHLH+BbHLH+DNA model, and (c) the CbHLH+BbHLH+PAS and CbHLH+BbHLH+PAS+DNA models. (TIF) [file pone.0155105.s002.tif]

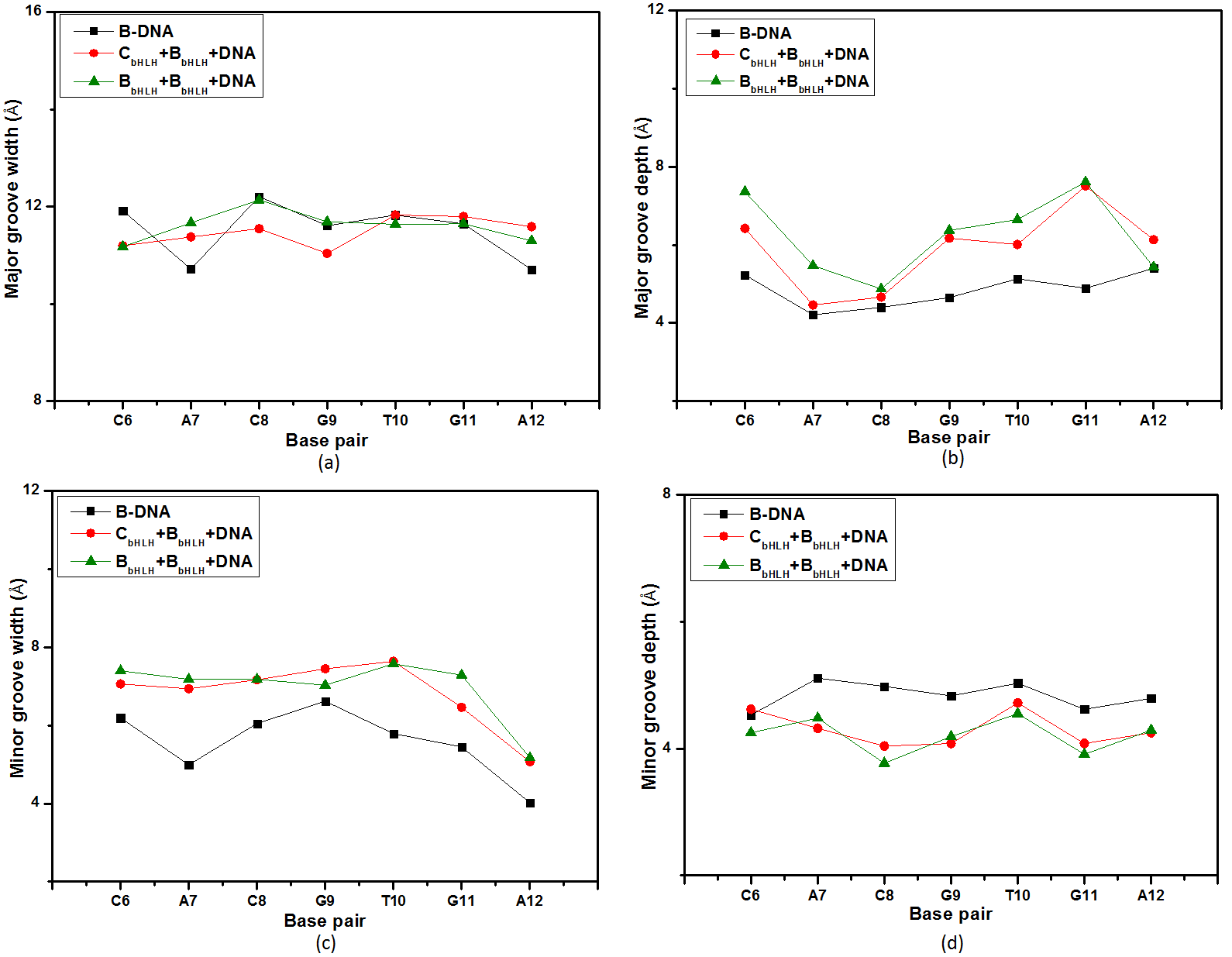

Supplement: S3 Fig — Groove widths and depths of the B-DNA (black line with square), CbHLH+BbHLH+DNA (red line with circle) and BbHLH+BbHLH+DNA (green line with up-triangle) models. (a) Major groove widths, (b) major groove depths, (c) minor groove widths and (d) minor groove depths for the time-averaged structures of DNA conformations. (TIF) [file pone.0155105.s003.tif]

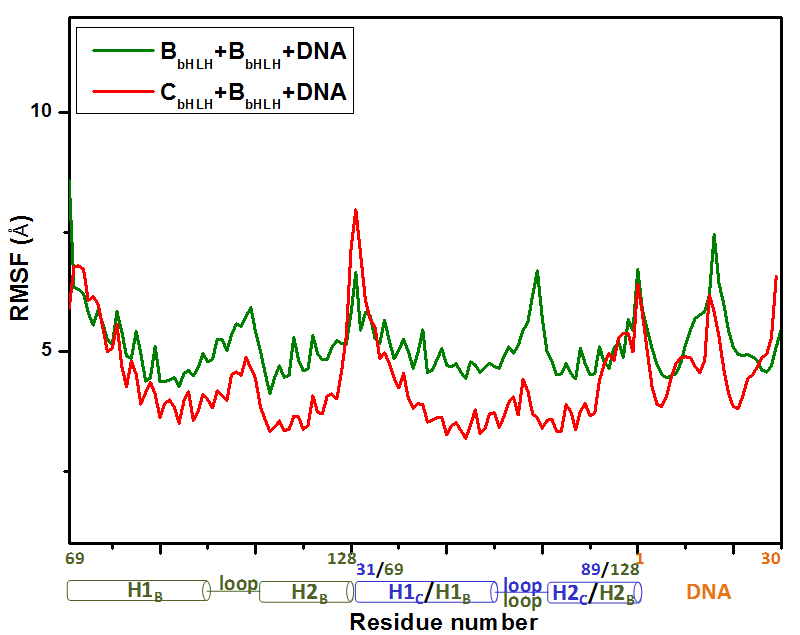

Supplement: S4 Fig — The fluctuations of residues and bases in the CbHLH+BbHLH+DNA (red) and BbHLH+BbHLH+DNA (green) models. (TIF) [file pone.0155105.s004.tif]

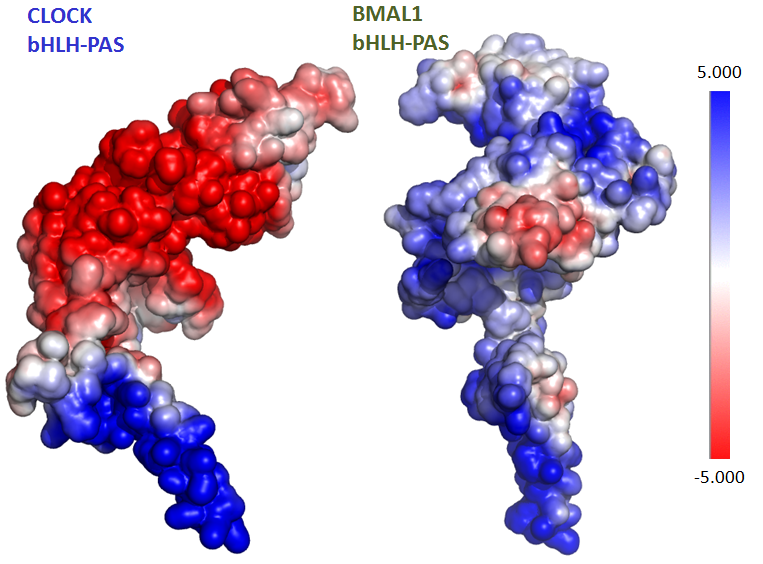

Supplement: S5 Fig — The electrostatic surface potentials for the bHLH-PAS domains of the CLOCK and BMAL1 proteins in the CbHLH+BbHLH+PAS+DNA model. (TIF) [file pone.0155105.s005.tif]

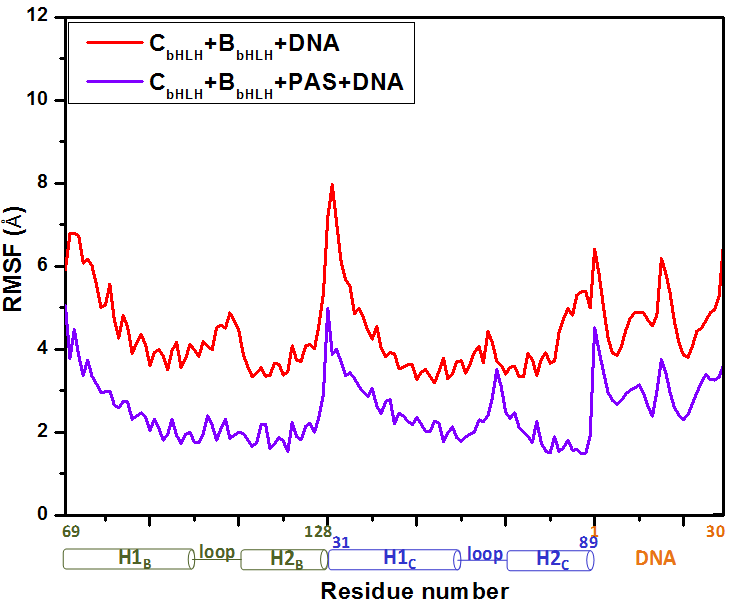

Supplement: S6 Fig — The fluctuations of residues and bases in the CbHLH+BbHLH+DNA (red) and CbHLH+BbHLH+PAS+DNA (violet) models. (TIF) [file pone.0155105.s006.tif]
