# Supplementary material for: Investigations of the CLOCK and BMAL1 Proteins Binding to DNA: A Molecular Dynamics Simulation Study
Source: PLoS One. 2016 May 6;11(5):e0155105. doi: 10.1371/journal.pone.0155105 (PMC4859532; doi:10.1371/journal.pone.0155105)
Supplement: S1 Table — (PDF) [file pone.0155105.s007.pdf]

S1 Table. Components of MM-PBSA binding free energies (kcal mol^{-1}) calculated from three independent MD simulations, and the average $\Delta G_{binding}$ with the error range for the C_{bHLH}+B_{bHLH} model.

Simulation No.	Simulation 1	Simulation 2	Simulation 3
ΔE_{ele}	929.54	898.32	993.08
ΔE_{vdw}	-106.90	-112.43	-98.10
ΔE_{int}	0.00	0.00	0.00
$\Delta G_{np/solv}$	-13.25	-13.36	-11.82
$\Delta G_{pb/solv}$	-899.63	-850.33	-963.74
ΔG_{np}	-120.21	-125.79	-109.92
ΔG_{pb}	43.16	47.99	41.16
ΔTS	-64.50	-63.01	-58.99
$\Delta H_{binding}$	-77.05	-77.80	-68.76
$\Delta G_{binding}$	-12.55	-14.79	-9.77
Average $\Delta G_{binding}$		-12.37 ±2.60	
