# Supplementary material for: Investigations of the CLOCK and BMAL1 Proteins Binding to DNA: A Molecular Dynamics Simulation Study
Source: PLoS One. 2016 May 6;11(5):e0155105. doi: 10.1371/journal.pone.0155105 (PMC4859532; doi:10.1371/journal.pone.0155105)
Supplement: S2 Table — (PDF) [file pone.0155105.s008.pdf]

S2 Table. The occupancies (%) of hydrogen bonds and hydrophobic interactions of protein-DNA and protein-protein in the C_{bHLH}+B_{bHLH}+DNA and B_{bHLH}+B_{bHLH}+DNA models.

	**Hydrogen bonds**		H1$_C$-H2$_B$	(Gln49)OE1···H-NH1(Arg116)	39
	**C_{bHLH}+B_{bHLH}+DNA**		H1$_B$- H2$_C$	(Arg82)NH2-H···OE2(Glu94)	64
DNA-H1$_C$	(G3)O3'···H-OG(Ser42)	32		(Arg82)NH2-H'···OE1(Glu94)	44
	(A4)O2P···H-OG(Ser42)	84		**B_{bHLH}+B_{bHLH}+DNA**	
	(C6)N4-H···O1(Glu43)	98	DNA-H1$_B$	(G11)O6···H-NE2(His77)	93
	(A7)N6-H···OE2(Glu43)	70		(G9)O6···H-NH2(Arg85)	92
	(A7)N7···H-NH1(Arg46)	50		(T10)O2P···H-OG(Ser78)	90
	(C23)O2P···H-NH2(Arg47)	99		(G11)O1P···H-NH2(Arg74)	80
	(C23)O2P···H-NE(Arg47)	97		(C21)N4-H···OE2(Glu81)	79
	(C23)O5' ···H-NH2(Arg39)	61		(G9)O6···H-NH1(Arg85)	68
	(G24)N7···H-NH1(Arg47)	82		(G9)N7···H-NH1(Arg85)	57
	(G24)O2P···H-ND2(Asn40)	69		(G11)O1P/O2P···H-NE(Arg74)	93
	(T25)O2P···H-NE/NH2(Arg36)	90		(A5)O2P···H-NH2(Arg84)	100
	(T25)O1P···H-NH2(Arg36)	45		(G24)O6···H-NH2(Arg85)	91
	(G26)O6···H-NE(Arg39)	39		(T25)O2P···H-OG(Ser78)	81
DNA-H1$_B$	(C6)O2P····H-NH2(Arg46)	34		(G26)O2P···H-NE(Arg74)	81
	(C8)O2P···H-NH2(Arg85)	92		(C6)N4-H···OE2(Glu81)	79
	(C8)O2P···H-NE(Arg85)	70		(G24)N7···H-NH1(Arg85)	78
	(C8)O5'···H-NH2(Arg85)	50		(G26)O1P-H···NH2(Arg74)	78
	(G9)N7···H-NH1(Arg85)	65		(G26)O6 ···H-NE2(His77)	66
	(G11)O6···H-NE(His77)	89		(G24)O6···H-NH1(Arg85)	58
	(G11)O2P···H-NE(Arg74)	37		(G26)N7···H-NE2(His77)	45
	(C21)O2P···H-NH2(Arg84)	100		(G26)O2P···H-NH2(Arg74)	36
	(C21)N4-H···OE1(Glu81)	83	H1$_B$-H2$_B$	(Arg126)NH1-H···O(Lys82)	60
	(A22)N6-H···OE2(Glu81)	95			
	**Hydrophobic interactions**			(Ile78)CG2···CD1(Leu95)	88
	**C_{bHLH}+B_{bHLH}+DNA**			(Ile78)CG2···CD1(Leu98)	90
DNA-H1$_B$	DNA(T20)C7···CG2(Ile80) BMAL1	85		**B_{bHLH}+B_{bHLH}+DNA**	
H1$_B$-H2$_C$	(Leu74)CD1···CB(Met88)	80	H1$_B$-H2$_B$	(Phe91)CB···CB(Leu115)	80
	(Leu74)CD1··· CG(Met88)	77		(Val119)CB···CD1(Leu98)	90
	(Leu74)CB···CB(Phe91)	100		(Met122)CG···CB(Leu98)	76
	(Ile78)CG2···CB(Leu95)	95			
