# Supplementary material for: Investigations of the CLOCK and BMAL1 Proteins Binding to DNA: A Molecular Dynamics Simulation Study
Source: PLoS One. 2016 May 6;11(5):e0155105. doi: 10.1371/journal.pone.0155105 (PMC4859532; doi:10.1371/journal.pone.0155105)
Supplement: S3 Table — (PDF) [file pone.0155105.s009.pdf]

S3 Table. Components of MM-PBSA free energies (kcal mol^{-1}) for the C_{bHLH}+B_{Phos}+DNA, B_{Phos}+B_{Phos}+DNA, C_{bHLH}+B_{bHLH}+PAS and C_{bHLH}+B_{bHLH}+PAS+DNA models.

	**C_{bHLH}+B_{Phos}+ DNA**	**B_{Phos}+B_{Phos}+ DNA**	**C_{bHLH}+B_{bHLH}+ PAS**	**C_{bHLH}+B_{bHLH}+PAS+DNA**	
Receptor	C_{bHLH}+B_{Phos}	B_{Phos}+B_{Phos}	C_{bHLH}+PAS	C_{bHLH}+PAS	C_{bHLH}+B_{bHLH} +PAS
Ligand	DNA	DNA	B_{bHLH}+PAS	B_{bHLH}+PAS	DNA
ΔE_{ele}	-9004.78	-6482.59	-2276.37	-1956.23	-8916.17
ΔE_{vdw}	-138.57	-124.85	-509.31	-518.60	-141.98
ΔE_{int}	0.00	0.00	0.00	0.00	0.00
$\Delta G_{np/solv}$	-18.75	-16.46	-59.73	-59.93	-19.47
$\Delta G_{pb/solv}$	8981.16	6470.14	2375.71	2087.43	8869.97
ΔG_{np}	-157.32	-141.31	-569.04	-578.53	-161.55
ΔG_{pb}	-4.88	4.01	159.06	191.14	-26.73
ΔTS	-106.97	-96.27	-57.17	-57.60	-105.19
$\Delta H_{binding}$	-162.02	-137.30	-409.98	-387.39	-188.18
$\Delta G_{binding}$	-55.05	-41.03	-352.81	-329.79	-82.99
