# Supplementary material for: Investigations of the CLOCK and BMAL1 Proteins Binding to DNA: A Molecular Dynamics Simulation Study
Source: PLoS One. 2016 May 6;11(5):e0155105. doi: 10.1371/journal.pone.0155105 (PMC4859532; doi:10.1371/journal.pone.0155105)
Supplement: S4 Table — (PDF) [file pone.0155105.s010.pdf]

S4 Table. The occupancies (%) of hydrogen bonds of protein-DNA in the C_{bHLH}+B_{Phos}+DNA and B_{Phos}+B_{Phos}+DNA models.

	**Hydrogen bonds**			(G11)O6···H-NE2(His77)	58
	**C_{bHLH}+B_{Phos}+DNA**			**B_{Phos}+B_{Phos}+DNA**	
DNA-H1$_{C}$	(C6)-N4-H···OE1(Glu43)	55	DNA-H1$_{B'}$	(C8)O2P···H-NH2(Arg85)	99
	(C6)O2P···H-NH2(Arg46)	97		(C8)O2P···H-NE(Arg85)	100
	(C23)O2P···H-NH2(Arg47)	99		(C8)O5'···H-NH2(Arg85)	73
	(C23)O2P···H-NE(Arg47)	77		(T10)O2P···H-NH2(Arg74)	97
	(C23)O5'···H-NH2(Arg47)	41		(G11)O1P···H-NE2(His77)	55
	(G24)O2P···H-ND2(Asn40)	60		(T20)O2P···H-NH2(Arg84)	99
	(G24)N7···H-NH2/1(Arg47)	56		(G24)N7···H-NH1(Arg85)	88
	(G26)O1P···H-NH2(Arg36)	46		(C6)O2P···H-NH1(Arg84)	100
	(G26)O2P···H-NE(Arg36)	44		(C6)N4···H-OE2(Glu81)	100
	(G26)O2P···H-NH1(Arg39)	41		(A7)N6···H-OE1(Glu81)	94
DNA-H1$_{B'}$	(C8)O2P···H-NH2(Arg85)	98		(C23)O2P···H-NH2(Arg85)	97
	(C8)O2P···H-NE(Arg85)	95		(C23)O2P···H-NE(Arg85)	91
	(C8)O5'···H-NH2(Arg85)	59		(G24)N7···H-NH1(Arg85)	88
	(G9)N7···H-NH1(Arg85)	38		(G26)O6 ···H-NE2(His77)	73
