# Supplementary material for: Investigations of the CLOCK and BMAL1 Proteins Binding to DNA: A Molecular Dynamics Simulation Study
Source: PLoS One. 2016 May 6;11(5):e0155105. doi: 10.1371/journal.pone.0155105 (PMC4859532; doi:10.1371/journal.pone.0155105)
Supplement: S5 Table — (PDF) [file pone.0155105.s011.pdf]

S5 Table. The occupancies (%) of hydrogen bonds of protein-DNA and protein-protein in the C_{bHLH}+B_{bHLH}+PAS+DNA model.

	Hydrogen bonds	
DNA-$H1_C$	(A4)O2P···H-OG(Ser42)	68
	(C6)N4-H···O1(Glu43)	92
	(C6)N4-H···OE2(Glu43)	61
	(C6)N4-H···OE1(Glu43)	56
	(A7)N6-H···OE2(Glu43)	33
	(C14)O3’···H-NH1(Arg46)	50
	(C23)O2P···H-NH2(Arg47)	95
	(C23)O2P···H-NE(Arg47)	92
	(C23)O5’ ···H-NH2(Arg47)	63
	(G24)O5’···H-ND2(Asn40)	39
	(G26)O2P···H-NH1/2(Arg36)	70
DNA-$H1_B$	(C8)O2P···H-NH2(Arg85)	88
	(C8)O2P···H-NE(Arg85)	78
	(G9)N7···H-NH1(Arg85)	64
	(T10)O2P···H-OG(Ser78)	56
	(G11)O6/O1P···H-NE(His77)	88
	(G11)N7···H-NE2(His77)	34
	(G11)O6···H-NE2(His77)	88
	(G11)O2P···H-NH2(Arg74)	81
	(C21)O2P···H-NH2(Arg84)	99
	(A22)N7···H-NH1(Arg84)	31
	(A22)N6-H···OE1(Glu81)	69
	(A22)N4-H···OE2(Glu81)	64
PAS_C-PAS_B	(Asn107)ND2-H···OD1(Asp144)	47
	(Glu108)OE1···H-NE1(Arg238)	61
	(Glu108)OE1···H-N(Ala135)	53
	(Glu108)OE2···H-NE1(Arg238)	52
	(Glu116)OE2···H-NE2(Arg244)	85
	(Glu116)OE1···H-NE2(Arg244)	77
	(Glu116)OE1···H-NE(Arg244)	70
	(Asp119)OD2···H-NH2(Arg319)	97
	(Asp119)OD1···H-NH1(Arg319)	84
	(Asp119)OD2···H’-NH1(Arg319)	52
	(Tyr184)OH-H···OD2(Arg153)	40
	(Gln190)OE1···H-NE2(His149)	82
	(Leu191)O···H-NH2(Arg153)	59
	(Gly217)O···H-NH1(Arg153)	55
	(Glu283)O ···H-ND2(Asn425)	96
	(Pro295)O···H-N(Gly242)	81
	(Gly298)O···H-OG(Ser211)	96
	(Ser308)OG-H···OE1(Glu432)	48
	(Tyr310)O··· H-OH (Tyr433)	100
	(Asp311)O···H-NH2(Arg343)	93
	(Tyr313)O···H-NH1(Arg343)	81
	(Lys344)O···H-NE2(Gln207)	88
	(Gly345)O···H-NH1(Arg244)	97
$loop_C$-PAS_B	(Asp99)O···H-OH(Tyr433)	99
PAS_C-$loop_B$	(Asp178)O···H-NE(Arg334)	96
PAS_C-$H2_B$	(Glu183)OE2···H-NH2(Arg126)	80
	(Glu183)OE2···H-NH1(Arg126)	80
	(Glu183)OE1···H-NH2(Arg126)	53
PAS_C-loop’$_B$	(Tyr210)N-H···O(Arg334)	88
Loop’$_C$-PAS_B	(Glu270)OE1···H-OG(Ser237)	59
	(Glu274)OE2···H-NH2(Arg218)	92
	(Glu274)OE2···H-NE(Arg218)	52
