# Supplementary material for: Investigations of the CLOCK and BMAL1 Proteins Binding to DNA: A Molecular Dynamics Simulation Study
Source: PLoS One. 2016 May 6;11(5):e0155105. doi: 10.1371/journal.pone.0155105 (PMC4859532; doi:10.1371/journal.pone.0155105)
Supplement: S1 Text — (PDF) [file pone.0155105.s012.pdf]

S1 Text

Molecular dynamics simulation protocols

For the phosphorylated C_{bHLH}+B_{Phos}+DNA and B_{Phos}+B_{Phos}+DNA models, the phosphorylated residue Ser(PO3) was modified by substituting the -PO_4 group for the -OH group in the residue Ser. The parameters of the phosphorylated residue Ser(PO3) were implemented into AMBER's library to generate a new library, which contains the information read by TLEAP module. TLEAP synthesizes default force field data together with the newly generated library to provide the parameters for energy terms such as angles, dihedrals, impropers, and non-bonded interactions of phosphorylated residue Ser(PO3) [1]. The phosphorylated C_{bHLH}+B_{Phos}+DNA and B_{Phos}+B_{Phos}+DNA models with the Ser(PO3)78 residue were then constructed by loading the new generated AMBER library using TLEAP module based on the structures of the C_{bHLH}+B_{bHLH}+DNA and B_{bHLH}+B_{bHLH}+DNA models, respectively.

All MD simulations were carried out using the AMBER9 package [2] with the force field parameters of parm99 [3, 4], parmbsc0 refinement [5] and gaff [6]. The protocol for all MD simulations is described herein as follows: (1) the systems were energetically minimized to remove unfavorable contacts. Four cycles of minimizations were performed with 5000 steps of each minimization and harmonic restraints on the bHLH and bHLH-PAS domains of the CLOCK and BMAL1 proteins, and DNA from 100 kcal mol^{-1} ·$Å^{-2}$, 75 kcal mol^{-1} ·$Å^{-2}$, 50 kcal mol^{-1} ·$Å^{-2}$ to 25 kcal mol^{-1} ·$Å^{-2}$, which means that the restraints were relaxed stepwisely by 25 kcal mol^{-1} ·$Å^{-2}$ per cycle. The fifth cycle consists of 10000 steps of unrestrained minimization before heating process. The cutoff distance used for the non-bonded interactions was 10 Å. The SHAKE algorithm [7] was used to restrain the bonds containing hydrogen atoms. (2) Each energy-minimized structure was heated over 120

ps from 0 to 300 K (with a temperature coupling of 0.2 ps), while the positions of the bHLH and bHLH-PAS domains of the CLOCK and BMAL1 proteins, and DNA were restrained with a small value of 25 kcal ·mol^{-1} ·Å^{-2}. The constant volume was maintained during the processes. (3) The unrestrained equilibration of 200 ps with constant pressure and temperature conditions was carried out for each system. The temperature and pressure were allowed to fluctuate around 300 K and 1 bar, respectively, with the corresponding coupling of 0.2 ps. For each simulation, an integration step of 2 fs was used. (4) Finally, production runs of 50 or 100 ns were carried out by following the same protocol. A time point after thermal equilibration of 200 ps in each simulation was selected as a starting point for data collection. During the production runs, 25000 or 50000 structures for a simulation were saved for post-processing by uniformly sampling the trajectory.

To test the structural convergence, three independent MD simulations for each of the three binary C_{bHLH}+B_{bHLH}, B_{bHLH}+B_{bHLH} and C_{bHLH}+C_{bHLH} models with the same initial structure and the starting velocities (usually randomly assigned) were performed. All the simulations for each binary model have converged to one structure after the corresponding courses of simulations. The root-mean-square deviation (RMSD) values of all heavy atoms referenced to the corresponding initial structure and the average structures for each of all tested systems were examined from three independent simulations. The corresponding RMSD values and the superposition of the average structures only for the C_{bHLH}+B_{bHLH} model are shown in S1(a) and (b) Figs, respectively. It can be seen that all the three independent simulations have reached equilibrium after 30 ns, and converged to one structure. This provides a good test of whether our MD simulations are capable of reaching a non-distinguishable one when simulations reach equilibrium.

References

1. Craft Jr JW, Legge GB. An AMBER/DYANA/MOLMOL phosphorylated amino acid library set and incorporation into NMR structure calculations. Journal of biomolecular NMR. 2005;33(1):15-24.
2. Case DA, Darden TA, Cheatham III TE, Simmerling CL, Wang J, Duke RE, et al. AMBER 9. University of California, San Francisco. 2006;45.
3. Duan Y, Wu C, Chowdhury S, Lee MC, Xiong G, Zhang W, et al. A point-charge force field for molecular mechanics simulations of proteins based on condensed-phase quantum mechanical calculations. Journal of computational chemistry. 2003;24(16):1999-2012.
4. Lee MC, Duan Y. Distinguish protein decoys by using a scoring function based on a new AMBER force field, short molecular dynamics simulations, and the generalized born solvent model. Proteins: Structure, Function, and Bioinformatics. 2004;55(3):620-34.
5. Pérez A, Marchán I, Svozil D, Sponer J, Cheatham TE, Laughton CA, et al. Refinement of the AMBER force field for nucleic acids: improving the description of α/γ conformers. Biophysical journal. 2007;92(11):3817-29.
6. Wang J, Wolf RM, Caldwell JW, Kollman PA, Case DA. Development and testing of a general amber force field. Journal of computational chemistry. 2004;25(9):1157-74.
7. Miyamoto S, Kollman PA. SETTLE: an analytical version of the SHAKE and RATTLE algorithm for rigid water models. Journal of computational chemistry. 1992;13(8):952-62.
