# Supplementary material for: Investigations of the CLOCK and BMAL1 Proteins Binding to DNA: A Molecular Dynamics Simulation Study
Source: PLoS One. 2016 May 6;11(5):e0155105. doi: 10.1371/journal.pone.0155105 (PMC4859532; doi:10.1371/journal.pone.0155105)
Supplement: S2 Text — (PDF) [file pone.0155105.s013.pdf]

# S2 Text

## MM-PBSA calculation for free energy

The binding free energy ($\Delta G_{binding}$) was computed through calculating the free energy differences of ligand, receptor and their complex as follows:

$$\Delta G_{binding} = G_{complex} - G_{ligand} - G_{receptor}$$

where $G_{complex}$, $G_{ligand}$, and $G_{receptors}$ are the free energies of complex, ligand and receptor, respectively. In MM-PBSA, the free energy ($G$) of each state is estimated from molecular mechanical energy $E_{MM}$, solvation free energy $G_{SOLV}$ and vibrational, rotational, and translational entropies $S$, respectively.

$$G = E_{MM} + G_{SOLV} - TS$$

$$E_{MM} = E_{int} + E_{vdw} + E_{ele}$$

$$G_{SOLV} = G_{pb/solv} + G_{np/solv}$$

Where T is the temperature; $E_{int}$ is internal energy, i.e. the sum of bond, angle, and dihedral energies; $E_{vdw}$ is van der Waals energy; $E_{ele}$ is electrostatic energy; $G_{SOLV}$ is the sum of electrostatic solvation free energy, $G_{pb/solv}$, and the non-polar salvation free energy, $G_{np/solv}$. Energetic post-process of single-trajectory was performed for each MM-PBSA calculation by using the MM-PBSA module of AMBER9 program through molecular mechanics and a continuum solvent model [1]. $G_{np/solv}$ is non-polar solvation free energy, which was calculated by using a solvent accessible surface area (SASA) as follows:

$$G_{np/solv} = \mathrm{rSASA} + \mathrm{b}$$

The SASA is the solvent-accessible surface area, and is estimated using Sanner's algorithm implemented in the Molsurf program in AMBER9 [2] with a probe radius of 1.4 Å. The surface tension proportionality constant (r) and the free energy of non-polar solvation for a point solute (b) are set to 0.00542 kcal $mol^{-1}$ $Å^{-2}$ and 0.92

kcal mol$^{-1}$, respectively. The entropy *S* is estimated by a normal mode analysis of the harmonic vibrational frequencies, calculated using the Nmode module in AMBER9 package [3]. Prior to the normal mode calculations, each structure was fully minimized using a distance dependent dielectric of ε = 4r (r is the distance between two atoms) to mimic the solvent dielectric change from the solute to solvent until the root-mean-square of the elements of the gradient vector was less than $5 \times 10^{-4}$ kcal mol$^{-1}$ ·Å$^{-1}$. Then, the entropy was calculated based on standard statistical mechanics expressions [4, 5]. Moreover, note that MM-PBSA free energies do not usually replicate the experimental free energy in absolute value. This approach provides reliable binding affinity ranking and exhibits good correlation with experiments [6-12]. Therefore, MM-PBSA only provides modest accuracy for relative binding affinities in systems dominated by electrostatics.

For each of the $C_{bHLH}$+$B_{bHLH}$, $B_{bHLH}$+$B_{bHLH}$, $C_{bHLH}$+$C_{bHLH}$, $C_{bHLH}$+$B_{bHLH}$+DNA, $B_{bHLH}$+$B_{bHLH}$+DNA, $C_{bHLH}$+$B_{bHLH}$+PAS and $C_{bHLH}$+$B_{bHLH}$+PAS+DNA models, the last 10ns trajectory of the production dynamics stage was used for binding free energy calculations of MM-PBSA, namely, the 1000 snapshots of each model at a 10-ps interval for computation of enthalpy and 20 snapshots at 500-ps intervals for computation of entropy. To verify the accuracy of the calculated energies, the protein-protein MM-PBSA binding free energies for the $C_{bHLH}$+$B_{bHLH}$ model were calculated by extracting the last 10ns trajectory from each of three independent MD simulations. The calculation details and the error range of the MM-PBSA binding free energy for the $C_{bHLH}$+$B_{bHLH}$ model are shown in S1 Table. It can be seen that the calculated MM-PBSA binding free energies present the certain calculated accuracy with the maximum error range of 2.60 kcal mol$^{-1}$ for this model. For each of the $C_{bHLH}$+$B_{Phos}$+DNA and $B_{Phos}$+$B_{Phos}$+DNA models, the last 20ns trajectory of the

production dynamics stage was used for the binding free energy calculations of MM-PBSA due to the unstable characteristics of the phosphorylated BMAL1 protein binding to DNA. Moreover, the bHLH domains and the DNA molecule for the $C_{bHLH}$+$B_{bHLH}$+PAS and $C_{bHLH}$+$B_{bHLH}$+PAS+DNA models were taken for the entropy calculations due to computational cost.

In addition, the MM-PBSA energy decompositions by per-residue for the $C_{bHLH}$+$B_{bHLH}$+DNA and $C_{bHLH}$+$B_{Phos}$+DNA models were performed to address the contribution of each residue to the binding free energies, which can provide a full description of energetic influences on binding affinity. Per-residue free energy decompositions were performed on the molecular mechanics, solvation free energies that were calculated by the generalized Born method, and surface-based energies. As Poisson-Boltzmann energies cannot be decomposed by residues, only results obtained with the generalized Born method are reported in this work [13].

## References


1. Case DA, Darden TA, Cheatham III TE, Simmerling CL, Wang J, Duke RE, et al. AMBER 9. University of California, San Francisco. 2006;45.
2. Connolly ML. Analytical molecular surface calculation. Journal of Applied Crystallography. 1983;16(5):548-58.
3. Kottalam J, Case DA. Langevin modes of macromolecules: applications to crambin and DNA hexamers. Biopolymers. 1990;29(10-11):1409-21.
4. Kollman PA, Massova I, Reyes C, Kuhn B, Huo S, Chong L, et al. Calculating structures and free energies of complex molecules: combining molecular mechanics and continuum models. Accounts of chemical research. 2000;33(12):889-97.
5. Frank J. Introduction to Computational Chemistry. Editorial Offices October. 1999.
6. Basdevant N, Weinstein H, Ceruso M. Thermodynamic basis for promiscuity and selectivity in protein-protein interactions: PDZ domains, a case study. Journal of the American Chemical Society. 2006;128(39):12766-77.
7. Gohlke H, Case DA. Converging free energy estimates: MM-PB (GB) SA studies on the protein-protein complex Ras–Raf. Journal of computational chemistry. 2004;25(2):238-50.
8. Grünberg R, Nilges M, Leckner J. Flexibility and conformational entropy in protein-protein binding. Structure. 2006;14(4):683-93.
9. Kuhn B, Kollman PA. Binding of a diverse set of ligands to avidin and streptavidin: an accurate quantitative prediction of their relative affinities by a combination of molecular mechanics and continuum solvent models. Journal of medicinal chemistry. 2000;43(20):3786-91.
10. Pearlman DA. Evaluating the molecular mechanics Poisson-Boltzmann surface area free energy method using a congeneric series of ligands to p38 MAP kinase. Journal of medicinal chemistry. 2005;48(24):7796-807.
11. Simard JR, Pawar V, Aust B, Wolf A, Rabiller M, Wulfert S, et al. High-throughput screening to identify inhibitors which stabilize inactive kinase conformations in p38α. Journal of the American Chemical Society. 2009;131(51):18478-88.
12. Wang J, Morin P, Wang W, Kollman PA. Use of MM-PBSA in reproducing the binding free energies to HIV-1 RT of TIBO derivatives and predicting the binding mode to HIV-1 RT of efavirenz by docking and MM-PBSA. Journal of the American Chemical Society. 2001;123(22):5221-30.
13. Gohlke H, Kiel C, Case DA. Insights into protein–protein binding by binding free energy calculation and free energy decomposition for the Ras–Raf and Ras–RalGDS complexes. Journal of molecular biology. 2003;330(4):891-913.
