# Supplementary material for: Investigations of the CLOCK and BMAL1 Proteins Binding to DNA: A Molecular Dynamics Simulation Study
Source: PLoS One. 2016 May 6;11(5):e0155105. doi: 10.1371/journal.pone.0155105 (PMC4859532; doi:10.1371/journal.pone.0155105)
Supplement: S3 Text — (PDF) [file pone.0155105.s014.pdf]

S3 Text

Analyses of fluctuation, correlation, interaction, interhelical angle/distance, and DNA groove parameter

The root-mean-square fluctuations (RMSF) values of residues/bases are a measure of fluctuations and flexibility of backbone Cα of protein/P atom of DNA over the trajectory broken down by residues/bases in comparison to the average structures [1, 2]. $RMSF_i$ of the Cα/P atom of each residue/base was calculated as follows:

$$RMSF_i = \sqrt{\frac{1}{T}\sum_{t=1}^{T}(r_i(t) - \langle r_i \rangle)^2} \qquad (1)$$

Where T is the number of snapshots considered in the time trajectory; $r_i(t)$ is the position of the Cα/P atom of residue/base i at time t; and $\langle r_i \rangle$ is the time-averaged position of the Cα/P atom of residue/base i. The dynamic feature of a protein/DNA and the extent of correlation of the motions of the different regions in a protein/DNA were assessed via the calculation of cross-correlation coefficients, $C(i, j)$ given as follows:

$$C(i, j) = \langle \Delta r_i \times \Delta r_j \rangle \big/ \left(\langle \Delta r_i^2 \rangle \langle \Delta r_j^2 \rangle \right)^{1/2} \qquad (2)$$

In the equation, Δr_i and Δr_j are the displacement vectors for atoms i and j, respectively, and the angle brackets denotes the ensemble average. In the present study, the correlation coefficients were averaged over the regions of the protein/DNA, and the resultant cross correlation coefficients are presented in the form of a two-dimensional graph. These analyses in the present work were calculated by using PTRAJ module in AMBER9 program [1].

A hydrogen bond and a hydrophobic contact are defined as a donor-acceptor distance of <3.5 Å and a donor-proton-acceptor angle of >120 ° [3, 4], and as a C-C distance of <4.5 Å [4] at -CH2-/-CH3 group with the occupancies of ≥30%, and ≥75%,

respectively. The percentages of occurrences of all possible hydrogen bonds and hydrophobic interactions were extracted from the MD trajectories using PTRAJ module in the AMBER9 program. To compare the amount of the total possible hydrogen bonds or hydrophobic interactions, the number of the total hydrogen bonds or the total hydrophobic interactions was calculated by defining one percent occupancy of hydrogen bond or hydrophobic interaction during the simulation equal to one number of hydrogen bond or hydrophobic interaction.

To analyze conformational changes in the relative orientations of any two helices, the program INTERHLX (written by Kyoko Yap, available at http://structbio.vanderbilt.edu/chazin/wisdom/interhel.html) was used to calculate the distances and the angles between the helices in the binary C_{bHLH}+B_{bHLH}, B_{bHLH}+B_{bHLH} and C_{bHLH}+C_{bHLH} models, and the ternary C_{bHLH}+B_{bHLH}+DNA and B_{bHLH}+B_{bHLH}+DNA models. The program was applied to calculate the sign of the angle between two helices by following this convenient role: The two helices are taken to be positioned by helix I being in front of helix II. Helix I (from N to C) is used to define first vertical vector. A second vertical vector is defined with its tail at the C-terminus of helix II. The angle between helices I and II is the rotation required to align the head of the second vector with the N-terminus of helix II. The vector is rotated in the direction that produces an angle of less than 180 degrees with the clockwise or counterclockwise rotation represented by positive or negative sign. This program can also provide other geometry-based parameters such as interhelical distances [5, 6].

The DNA groove parameters from the trajectories of simulations for the C_{bHLH}+B_{bHLH}+DNA, B_{bHLH}+B_{bHLH}+DNA models and a canonical B-DNA were calculated using the CURVES program to investigate the disturbance of DNA. The

PTRAJ module of AMBER9 program was used to extract the production conformations. These extracted time-averaged structures were saved in the Protein Data Bank (PDB) format. Each nucleotide type was converted from the AMBER format to PDB format, and the resulting snapshots were submitted to the CURVES program. The following CURVES parameters were extracted, i.e. major groove width, major groove depth, minor groove width and minor groove depth [7].

References

1. Case DA, Darden TA, Cheatham III TE, Simmerling CL, Wang J, Duke RE, et al. AMBER 9. University of California, San Francisco. 2006;45.
2. Sadiq SK, De Fabritiis G. Explicit solvent dynamics and energetics of HIV-1 protease flap opening and closing. Proteins: Structure, Function, and Bioinformatics. 2010;78(14):2873-85.
3. Brocklehurst SM, Perham RN. Prediction of the three-dimensional structures of the biotinylated domain from yeast pyruvate carboxylase and of the lipoylated H-protein from the pea leaf glycine cleavage system: a new automated method for the prediction of protein tertiary structure. Protein Science : A Publication of the Protein Society. 1993;2(4):626-39.
4. Chuprina VP, Rullmann JAC, Lamerichs RMJN, van Boom JH, Boelens R, Kaptein R. Structure of the Complex of lac Repressor Headpiece and an 11 Base-pair Half-operator Determined by Nuclear Magnetic Resonance Spectroscopy and Restrained Molecular Dynamics. Journal of Molecular Biology. 1993;234(2):446-62.
5. Yap KL, Ames JB, Swindells MB, Ikura M. Diversity of conformational states and changes within the EF-hand protein superfamily. Proteins: Structure, Function, and Bioinformatics. 1999;37(3):499-507.
6. Yap KL, Ames JB, Swindells MB, Ikura M. Vector Geometry Mapping. Calcium-Binding Protein Protocols: Volume 2: Methods and Techniques: Springer; 2002. p. 317-24.
7. Lavery R, Sklenar H. The definition of generalized helicoidal parameters and of axis curvature for irregular nucleic acids. Journal of Biomolecular Structure and Dynamics. 1988;6(1):63-91.
